# Evidence for the Involvement of Loosely Bound Plastosemiquinones in Superoxide Anion Radical Production in Photosystem II

**DOI:** 10.1371/journal.pone.0115466

**Published:** 2014-12-26

**Authors:** Deepak Kumar Yadav, Ankush Prasad, Jerzy Kruk, Pavel Pospíšil

**Affiliations:** 1 Department of Biophysics, Centre of the Region Haná for Biotechnological and Agricultural Research, Faculty of Science, Palacký University, Olomouc, Czech Republic; 2 Department of Plant Physiology and Biochemistry, Faculty of Biochemistry, Biophysics and Biotechnology, Jagiellonian University, Kraków, Poland; National Research Council of Italy, ITALY

## Abstract

Recent evidence has indicated the presence of novel plastoquinone-binding sites, Q_C_ and Q_D_, in photosystem II (PSII). Here, we investigated the potential involvement of loosely bound plastosemiquinones in superoxide anion radical (O_2_^•−^) formation in spinach PSII membranes using electron paramagnetic resonance (EPR) spin-trapping spectroscopy. Illumination of PSII membranes in the presence of the spin trap EMPO (5-(ethoxycarbonyl)-5-methyl-1-pyrroline N-oxide) resulted in the formation of O_2_^•−^, which was monitored by the appearance of EMPO-OOH adduct EPR signal. Addition of exogenous short-chain plastoquinone to PSII membranes markedly enhanced the EMPO-OOH adduct EPR signal. Both in the unsupplemented and plastoquinone-supplemented PSII membranes, the EMPO-OOH adduct EPR signal was suppressed by 50% when the urea-type herbicide DCMU (3-(3,4-dichlorophenyl)-1,1-dimethylurea) was bound at the Q_B_ site. However, the EMPO-OOH adduct EPR signal was enhanced by binding of the phenolic-type herbicide dinoseb (2,4-dinitro-6-sec-butylphenol) at the Q_D_ site. Both in the unsupplemented and plastoquinone-supplemented PSII membranes, DCMU and dinoseb inhibited photoreduction of the high-potential form of cytochrome *b*_559_ (cyt *b*_559_). Based on these results, we propose that O_2_^•−^ is formed via the reduction of molecular oxygen by plastosemiquinones formed through one-electron reduction of plastoquinone at the Q_B_ site and one-electron oxidation of plastoquinol by cyt *b*_559_ at the Q_C_ site. On the contrary, the involvement of a plastosemiquinone formed via the one-electron oxidation of plastoquinol by cyt *b*_559_ at the Q_D_ site seems to be ambiguous. In spite of the fact that the existence of Q_C_ and Q_D_ sites is not generally accepted yet, the present study provided more spectroscopic data on the potential functional role of these new plastoquinone-binding sites.

## Introduction

Photosystem (PSII) is a heterodimeric multiprotein-pigment complex embedded in the thylakoid membrane of photosynthetic organisms such as cyanobacteria, algae and higher plants. Recent X-ray crystallographic structural analyses of PSII from the cyanobacteria *Thermosynechococcus elongatus* and *Thermosynechococcus vulcanus* demonstrated that PSII consists of 20 protein subunits, 35 chlorophylls, 12 carotenoids and 25 lipids per monomer [1–3]. During oxygenic photosynthesis, PSII functions as a water-plastoquinone oxidoreductase that oxidizes water to molecular oxygen and reduces plastoquinone to plastoquinol [4–5]. In these reactions, four electrons extracted from water by a water-splitting manganese complex on the electron donor side of PSII are transferred to the primary and secondary electron acceptors on the electron acceptor side of PSII [6–9]. It is well established that the primary and secondary electron acceptors are plastoquinones tightly and loosely bound to the Q_A_ and Q_B_ sites, respectively. One-electron reduction of plastoquinone at the Q_B_ site forms plastosemiquinone (Q_B_^•−^), which is subsequently stabilized by the protonation of proximal amino acid side chains (Q_B_H^•^), whereas the sequential one-electron reduction and protonation of Q_B_H^•^ forms plastoquinol (Q_B_H_2_).

Several biochemical studies have suggested that PSII contains two plastoquinone-binding sites in addition to the Q_A_ and Q_B_ sites [10–12]. Based on the study on photoreduction of cytochrome *b*_559_ (cyt *b*_559_) in the presence of exogenous plastoquinone, a third plastoquinone-binding site referred to as Q_C_ was proposed to be located closed to cyt *b*_559_ [10]. Later, the effects of herbicides and ADRY agents on the redox properties of cyt *b*_559_ provided more biochemical data on the existence of Q_C_ site [11–12]. Consistent with biochemical studies, the crystal structure of PSII at 2.9 Å resolution revealed the existence of Q_C_ site [2]. However, the Q_C_ site was not reported in the most recent PSII crystal structure at 1.9 Å resolution [3]. Hasegawa and Noguchi proposed that the affinity of plastoquinone to the Q_C_ site is lower compared to the Q_B_ site [13]. In agreement with this proposal, it has been recently suggested that ambiguity in the existence of Q_C_ site might be due to the different purification and crystallization procedures [14]. Recently, Kaminskaya and Shuvalov [15] identified a fourth plastoquinone-binding site denoted as Q_D_. The authors concluded that the Q_C_ site depicted in the PSII crystal structure is in a highly hydrophobic environment, while the Q_D_ site is located in a more polar environment. The urea-type herbicide DCMU (3-(3,4-dichlorophenyl)-1,1-dimethylurea) blocks Q_B_ to Q_B_^•−^ reduction at the Q_B_ site, whereas the phenolic-type herbicide dinoseb (2,4-dinitro-6-sec-butylphenol) prevents the oxidation of plastoquinol (Q_D_H_2_) to plastosemiquinone (Q_D_H^•^) by cyt *b*_559_ at the Q_D_ site [15].

The limitations on electron transport both on the electron donor and acceptor sides of PSII are associated with the formation of reactive oxygen species (ROS) [16–19]. Under high-light conditions, when light absorption by chlorophylls exceeds the utilization of excitation energy, the over-reduction of the electron acceptor side of PSII leads to leakage of electrons to molecular oxygen. The reduction of molecular oxygen results in the formation of superoxide anion radical (O_2_^•−^), which either spontaneously dismutates to hydrogen peroxide (H_2_O_2_) or forms bound peroxide through interactions with the non-heme [20] or heme iron in cyt *b*_559_ [21]. Subsequent reductions of either H_2_O_2_ by free metals or bound peroxide by the non-heme iron forms hydroxyl radicals (HO^•^) [20].

Several studies have demonstrated that various cofactors on the electron acceptor side of PSII can reduce molecular oxygen, forming O_2_^•−^. These cofactors are highly reducing species with a midpoint redox potential lower than the standard redox potential of the O_2_/O_2_^•−^ redox couple (*E*_0_ *´* = – 160 mV, pH 7). Molecular oxygen may be reduced by pheophytin (Pheo^•−^) [20, 22], the tightly bound plastosemiquinone at the Q_A_ site (Q_A_^•−^) [23], the loosely bound plastosemiquinone at the Q_B_ site (Q_B_^•−^) [24], free plastosemiquinone (PQ^•−^) [25] and the ferrous heme iron in the low-potential (LP) form of cyt *b*_559_ [26].

Due to a highly negative redox potential (*E*m (Pheo/Pheo^•−^) = – 505 to –610 mV, pH 6.5 to 7) [27–28], the reduction of molecular oxygen by Pheo^•−^ is likely. The favorable thermodynamic properties for reduction of molecular oxygen by Pheo^•−^ are limited by kinetic restrictions. Forward electron transport from Pheo^•−^ to Q_A_^•−^ is much more rapid than diffusion-limited reduction of molecular oxygen; thus, the reduction of molecular oxygen by Pheo^•−^ is less likely. However, under certain circumstances, such as limitation of electron transport from Pheo^•−^ to Q_A_^•−^, the Pheo^•−^ lifetime is prolonged, and the reduction of molecular oxygen is more likely.

In contrast to Pheo^•−^, reduction of molecular oxygen by Q_A_^•−^ and Q_B_^•−^ is less favorable from a thermodynamic perspective. In principle, the midpoint redox potentials of the Q_A_/Q_A_^•−^ (*E*m = – 60 to –140 mV, pH 7) [29–30] and Q_B_/Q_B_^•−^ (*E*m = – 45 mV, pH 7) [31] redox couples are greater than the standard redox potential of the O_2_/O_2_^•−^ redox couple (*E*_0_ *´* = – 160 mV, pH 7) [32]. When the concentrations of reactant (O_2_ ~ hundreds μM) and product (O_2_^•−^ ~ hundreds nM) differ, the operational redox potential of the O_2_/O_2_^•−^ redox couple is shifted to 0 mV or even positive values based on the Nernst equation [16–17]. Thus, the reduction of molecular oxygen by Q_A_^•−^ and Q_B_^•−^ seems to be more thermodynamically feasible. From a kinetic perspective, the lifetimes of Q_A_^•−^ and Q_B_^•−^ are sufficiently long for the diffusion-limited reduction of molecular oxygen. In addition to Q_A_^•−^ and Q_B_^•−^, free PQ^•−^ can reduce molecular oxygen (*E*m = –170 mV, pH 7) [31]; however, the probability of its formation by the interaction of free plastoquinone and free plastoquinol is very low [25]. It has been proposed that the reduction of molecular oxygen by ferrous heme iron in the LP form of cyt *b*_559_ produces O_2_^•−^ and may be thermodynamically feasible because the LP form of cyt *b*_559_ has a low midpoint redox potential (*E*m = -40 to +80 mV, pH 7) [21, 26, 33].

Herein, we studied whether loosely bound plastosemiquinones are involved in the light-induced O_2_^•−^ formation in PSII membranes using an electron paramagnetic resonance (EPR) spin-trapping spectroscopy. We provide evidence that O_2_^•−^ is produced via one-electron reduction of molecular oxygen by plastosemiquinones, which are formed through one-electron reduction of plastoquinone at the Q_B_ site (Q_B_^•−^) and one-electron oxidation of plastoquinol by cyt *b*_559_ at the Q_C_ site (Q_C_H^•^). By contrast, a role of plastosemiquinone formed at the Q_D_ site (Q_D_H^•^) in O_2_^•−^ formation is ambiguous.

## Materials and Methods

### 1. PSII membrane preparation

PSII membranes were isolated from fresh spinach leaves using the method reported previously by Berthold *et al*. [34] with modifications described by Ford and Evans [35]. The isolated PSII membranes were dissolved in a buffer solution containing 400 mM sucrose, 10 mM NaCl, 5 mM CaCl_2,_ 5 mM MgCl_2_ and 50 mM Mes-NaOH (pH 6.5) and stored at -80°C until further use. For PQ-supplemented PSII membranes, exogenous short-chain platoquinone containing one isoprenoid units in the side-chain (PQ-1) was added to the PSII membranes prior to illumination. 30 μM PQ-1 was added to the PSII membranes as an ethanol solution (the final concentration of ethanol did not exceed 1%).

### 2. EPR spin-trapping spectroscopy

O_2_^•−^ was detected by EPR spin-trapping spectroscopy using EMPO (5-(ethoxycarbonyl)-5-methyl-1-pyrroline N-oxide; Alexis Biochemicals, Lausen, Switzerland) as the spin trap. PSII membranes (150 μg Chl ml^-1^) were illuminated with a continuous white light (1000 μmol photons m^-2^ s^-1^) in a glass capillary tube (Blaubrand intraMARK, Brand, Germany) with 25 mM EMPO, 100 μM Desferal and 40 mM MES buffer (pH 6.5). PSII membranes were illuminated using a halogen lamp with a light guide (Schott KL 1500, Schott AG, Mainz, Germany) at room temperature. The spectra were recorded using an EPR spectrometer Mini Scope MS400 (Magnettech GmbH, Germany). The following EPR conditions were used: microwave power, 10 mW; modulation amplitude, 1 G; modulation frequency, 100 kHz; sweep width, 100 G; and scan rate, 1.62 G s^-1^. For quantification, intensity of EPR signal was evaluated as the relative height of peak of the first derivative of the EPR absorption spectrum.

### 3. Optical measurements

The redox properties of cyt *b*_559_ were studied using an Olis RSM 1000 spectrometer (Olis Inc., Bogart, Georgia, USA). The redox states of cyt *b*_559_ in PSII membranes (150 μg Chl ml^-1^) were determined based on the changes in the absorbance at 559 nm upon stepwise additions of 50 μM potassium ferricyanide, 8 mM hydroquinone, 5 mM sodium ascorbate and sodium dithionite in a cuvette at room temperature using the method in Tiwari and Pospíšil [21] with certain modification. The redox forms of cyt *b*_559_ in the PSII membranes were determined by subtracting the control from the treatment spectra: for the HP form of cyt *b*_559_, the hydroquinone-reduced spectra were subtracted from the ferricyanide-oxidized cyt *b*_559_; for the IP form of cyt *b*_559_, the ascorbate-reduced spectra were subtracted from the hydroquinone-reduced cyt *b*_559_; and for the LP form of cyt *b*_559_, the dithionite-reduced spectra were subtracted from the ascorbate-reduced cyt *b*_559_. In photoreduction measurements, the photoreduced HP form of cyt *b*_559_ (PH) was calculated based on the difference between the absorbance spectra measured after illumination for 180 s and the dark-adapted ferricyanide oxidized spectrum and hydroquinone-reduced spectra were subtracted from photoreduced HP form of cyt *b*_559_ to get unreduced HP form of cyt *b*_559_. The PSII membranes were illuminated with continuous white light (1000 μmol photons m^-2^ s^-1^) in the cuvette, which was rotated by 90° at intervals of 15 s.

### 4. High-pressure liquid chromatography

The loosely bound plastoquinone was measured using the method in Wydrzynski and Inoue [36]. A 1 ml aliquot of the PSII membranes (300 μg Chl ml^-1^) was mixed with 3 ml of heptane and 30 μl of isobutanol, followed by vortexing for 1 h in the dark. The mixture was then centrifuged at 4000 x *g* for 10 min. The plastoquinone content in the upper organic layer was determined by HPLC based on the method of Kruk and Karpinski [37].

## Results

### 1. Superoxide anion radical production in unsupplemented PSII membranes

**Fig 1:**
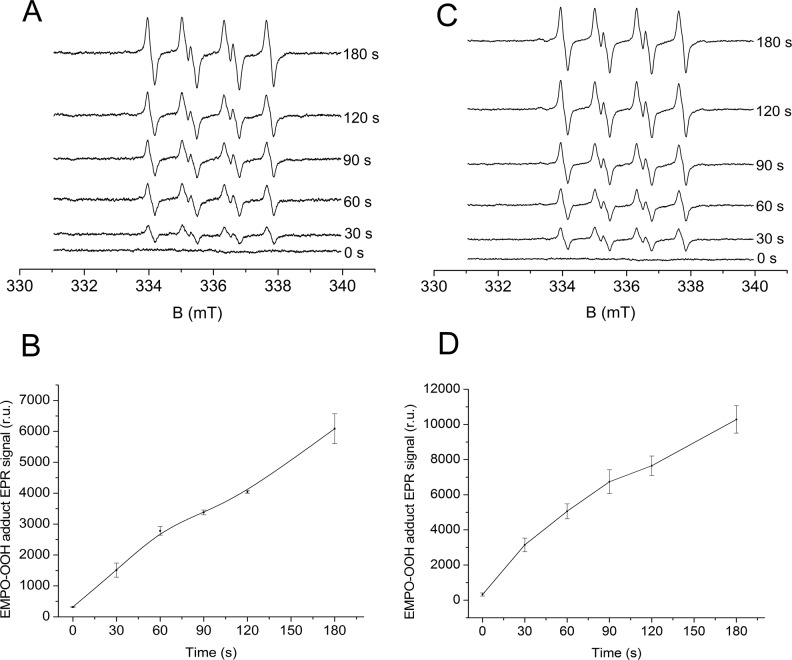
Light-induced EMPO-OOH adduct EPR spectra measured using unsupplemented and PQ-supplemented PSII membranes. EMPO-OOH adduct EPR spectra were obtained after illumination of PSII membranes (150 μg Chl ml^-1^) with white light (1000 μmol photons m^-2^ s^-1^) in the absence [A, B] and presence of exogenous PQ-1 [C, D] and in the presence of 25 mM EMPO, 100 μM Desferal and 40 mM MES (pH 6.5). Figures B and D shows mean ± SD, where n = 3. 30 μM PQ-1 was added to PSII membranes prior to illumination.

**Fig 2:**
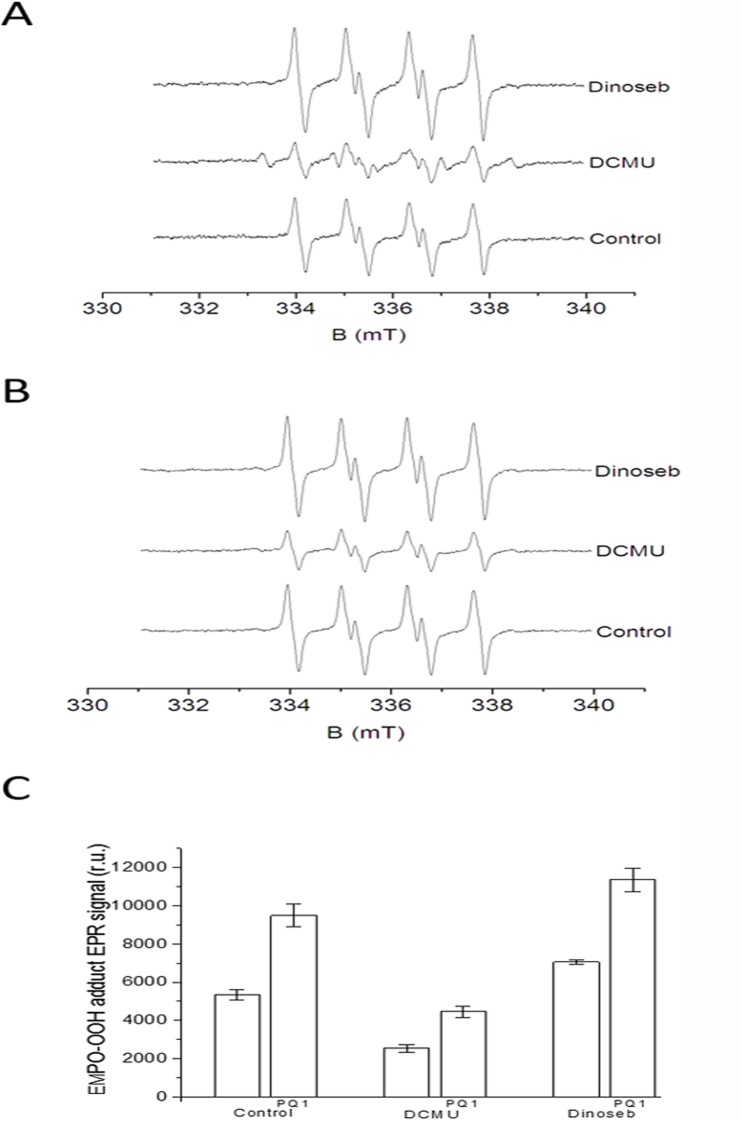
The effects of DCMU and dinoseb on EMPO-OOH adduct EPR spectra measured using unsupplemented and PQ-supplemented PSII membranes. EMPO-OOH adduct EPR spectra were measured using unsupplemented [A] and PQ-supplemented PSII membranes [B] in the presence of DCMU and dinoseb. Prior to illumination, DCMU (20 μM) and dinoseb (200 μM) were added to the membranes. [C] The relative intensity (mean ± SD, n = 3) of the light-induced EMPO-OOH adduct EPR signal measured using unsupplemented and PQ-supplemented PSII membranes. The other experimental conditions were the same as described in Fig. 1.

Light-induced O_2_^•−^ formation in the unsupplemented PSII membranes was measured using EPR spin-trapping spectroscopy. For spin-trapping, we used the spin trap compound EMPO, which reacts with O_2_^•−^ to form an EMPO-OOH adduct [38]. No EMPO-OOH adduct EPR signal appeared immediately after addition of EMPO to the unsupplemented PSII membranes in the dark (Fig. 1A). Illumination of the unsupplemented PSII membranes in the presence of EMPO resulted in the production of an EMPO-OOH adduct EPR signal (Fig. 1A). To prevent EMPO-OH adduct formation, the strong iron chelator Desferal was used to decrease the level of free iron available to produce HO^•^ through the Fenton reaction [26, 39]. Fig. 2B shows the time profile for the EMPO-OOH adduct EPR signal measured for the unsupplemented PSII membranes. These results demonstrate that the illumination of unsupplemented PSII membranes results in the formation of O_2_^•−^.

### 2. Superoxide anion radical production in PQ-supplemented PSII membranes

To study the role of loosely bound plastosemiquinone in O_2_^•−^ formation, light-induced O_2_^•−^ formation was measured in the presence of exogenous PQ-1. Because PQ-1 is smaller than the natural molecule PQ-9, PQ-1 can better penetrate the membrane and substitute for PQ-9 as an electron acceptor in PSII. The observation that the addition of PQ-1 to EMPO did not generate any EPMO-OOH adduct EPR spectrum indicates that PQ-1 does not directly interact with EMPO (data not shown). In the dark, the addition of PQ-1 to the PSII membranes in the presence of EMPO did not produce an EPR signal; however, exposure of PQ-supplemented PSII membranes to white light resulted in the formation of an EMPO-OOH adduct EPR signal (Fig. 1C). The time profile of the EMPO-OOH adduct EPR signal measured after addition of exogenous PQ-1 to the PSII membranes revealed that the intensity of the EMPO-OOH adduct EPR signal was enhanced by 70% as compared to unsupplemented PSII membranes (Fig. 1D). These results indicate that plastosemiquinones are involved in light-induced O_2_^•−^ production in PSII.

### 3. The effects of DCMU and dinoseb on superoxide anion radical production in unsupplemented PSII membranes

To investigate where loosely bound plastosemiquinones involved in O_2_^•−^ production are formed, the effects of two herbicides, DCMU (bound at the Q_B_ site) and dinoseb (bound at the Q_D_ site) on the EMPO-OOH adduct EPR signal were studied in the unsupplemented PSII membranes. When the unsupplemented PSII membranes were illuminated in the presence of DCMU, the EMPO-OOH adduct EPR signal was suppressed by 50%, whereas the remaining EPMO-OOH EPR signal (50%) was insensitive to DCMU (Fig. 2A and C). In previous studies [24, 26, 40, 41], the relative proportion of DCMU-sensitive and DCMU-insensitive O_2_^•−^ production in PSII varied, likely due to the endogenous platoquinone content. In addition to the EMPO-OOH adduct EPR signal, the EPR spectrum measured in the presence of DCMU comprises an EMPO-R adduct EPR signal formed by the interaction between EMPO and a carbon-centered radical, the origin of which is unknown. These observations reveal that 1) the DCMU-sensitive EMPO-OOH adduct EPR signal corresponds to O_2_^•−^ formed at or after the Q_B_ site (i.e., reduction of molecular oxygen by loosely bound plastosemiquinones formed by one-electron reduction of plastoquinone and one-electron oxidation of plastoquinol) and 2) the DCMU-insensitive EMPO-OOH adduct EPR signal corresponds to O_2_^•−^, which is formed before the Q_B_ site (i.e., reduction of molecular oxygen by Pheo^•−^ and Q_A_^•−^). When dinoseb was added to the unsupplemented PSII membranes prior to illumination, the EMPO-OOH adduct EPR signal was enhanced by 25% (Fig. 2A and C). Due to the fact that the occupation of the Q_D_ site does not eliminate O_2_^•−^ production, the production of O_2_^•−^ by reduction of molecular oxygen by plastosemiquinone at the Q_D_ site is ambiguous.

### 4. The effects of DCMU and dinoseb on superoxide anion radical production in PQ-supplemented PSII membranes

Addition of DCMU to PQ-supplemented PSII membranes decreased the EMPO-OOH adduct EPR signal by 55% (Fig. 2B and C). Similar to unsupplemented PSII membranes, in PQ-supplemented PSII membranes, 1) O_2_^•−^ is formed at or after the Q_B_ site via reduction of molecular oxygen by plastosemiquinone formed via one-electron reduction of plastoquinone and one-electron oxidation of plastoquinone and 2) O_2_^•−^ is formed prior to the Q_B_ site by reduction of molecular oxygen by Pheo^•−^ and Q_A_^•−^. The intensity of the EMPO-OOH adduct EPR signal after the addition of DCMU was higher for the PQ-supplemented PSII membranes than for the unsupplemented PSII membranes (Fig. 2C). When dinoseb was added to the PQ-supplemented PSII membranes prior to illumination, the EMPO-OOH adduct EPR signal was enhanced by 17% (Fig. 2B and C). The intensity of the EMPO-OOH adduct EPR signal after the addition of dinoseb was higher for PQ-supplemented PSII membranes compared to unsupplemented PSII membranes (Fig. 2C). Similar to the unsupplemented PSII membranes, the effect of dinoseb on O_2_^•−^ production in PQ-supplemented PSII membranes indicate that the Q_D_ site is unlikely involved in O_2_^•−^ production.

### 5. Different redox forms of cyt b_559_ in the unsupplemented and PQ-supplemented PSII membranes

**Fig 3:**
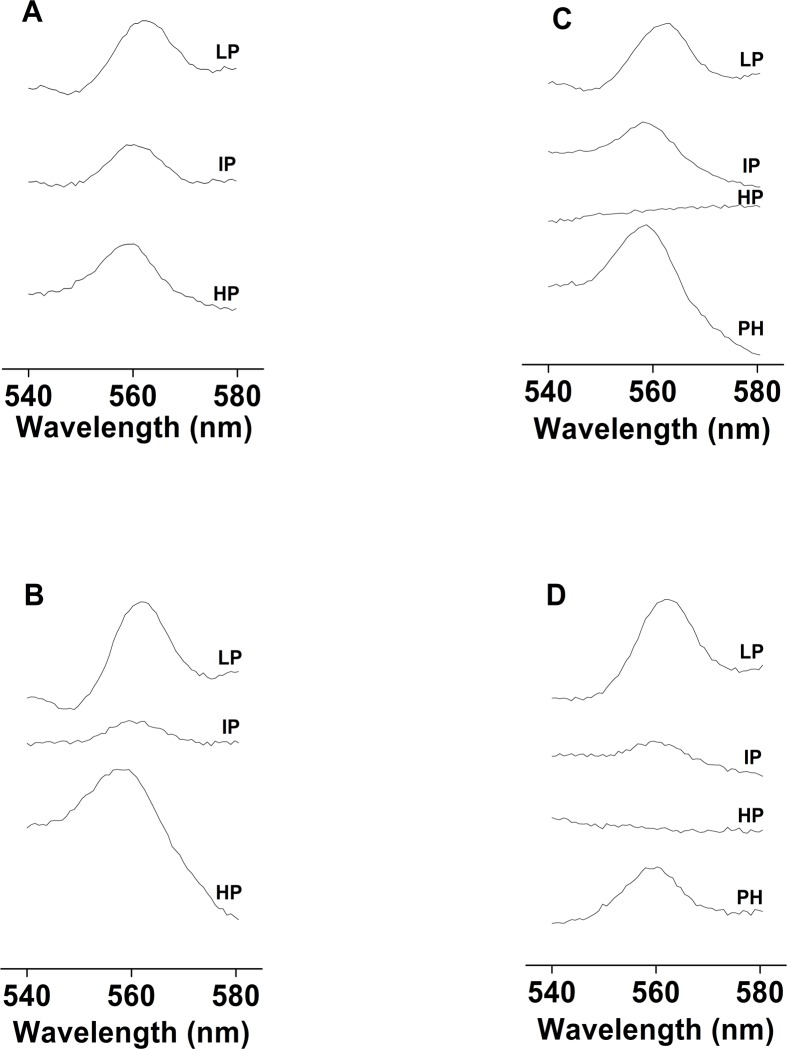
Differences in redox spectra and cyt *b*_559_ photoreduction measured using unsupplemented and PQ-supplemented PSII membranes. Differences in the redox spectra of cyt *b*_559_ measured in the dark using unsupplemented [A] and PQ-supplemented PSII membranes [B]. 100 μM PQ-1 was added to the PSII membranes prior to the experiments. To measure cyt *b*_559_ photoreduction, unsupplemented [C] and PQ-supplemented PSII membranes [D] were illuminated for 180 s at high light intensity (1000 μmol photons m^-2^ s^-1^). The spectra represent the difference in the light minus ferricyanide-oxidized spectra [the photoreduced HP form of cyt *b*_559_, (PH)], hydroquinone-reduced minus ferricyanide-oxidized or hydroquinone-reduced minus light spectra [HP form of cyt *b*_559_, (HP)], ascorbate-reduced minus hydroquinone-reduced spectra [IP form of cyt *b*_559_, (IP)] and dithionite-reduced minus ascorbate-reduced spectra [LP form of cyt *b*_559_, (LP)].

To determine the different redox forms of cyt *b*_559_, we measured changes in absorption at 559 nm in the unsupplemented and PQ-supplemented PSII membranes. The different redox forms of cyt *b*_559_ were discerned by examining the hydroquinone-reduced minus ferricyanide-oxidized (HP) spectra, ascorbate-reduced minus hydroquinone-reduced (IP) spectra, and dithionite-reduced minus ascorbate-reduced (LP) spectra. In the unsupplemented PSII membranes, 40% of cyt *b*_559_ was in the hydroquinone-reducible HP form, 22% was in the sodium ascorbate-reducible IP form, and 38% was in the dithionite-reducible LP form (Fig. 3A). In the supplemented PSII membranes, the levels of the hydroquinone-reducible HP, sodium ascorbate-reducible IP and dithionite-reducible LP forms of cyt *b*_559_ were 42, 12 and 46% (Fig. 3B). These observations confirm the presence of the HP, IP and LP forms of cyt *b*_559_ in both unsupplemented and PQ-supplemented PSII membranes.

### 6. Cyt b_559_ photoreduction in the unsupplemented and PQ-supplemented PSII membranes

To observe the light-induced reducible redox form of cyt *b*_559_, cyt *b*_559_ photoreduction was measured in both unsupplemented and PQ-supplemented PSII membranes. When the unsupplemented PSII membranes were exposed to white light, the HP form of cyt *b*_559_ was reduced (Fig. 3C). Addition of hydroquinone to the unsupplemented PSII membranes after illumination did not further reduce the HP form of cyt *b*_559_; however, addition of sodium ascorbate and sodium dithionite reduced the IP and LP forms of cyt *b*_559_ (Fig. 3C). Similarly, exposure of PQ-supplemented PSII membranes to white light reduced the HP form of cyt *b*_559_ (Fig. 3D); however, addition of hydroquinone to PQ-supplemented PSII membranes after illumination did not further reduce the HP form. Addition of ascorbate and dithionite to PQ-supplemented PSII membranes reduced the IP and LP forms of cyt *b*_559_ (Fig. 3D). These results demonstrate that illumination of the unsupplemented and PQ-supplemented PSII membranes reduced the HP form of cyt *b*_559_.

### 7. The effects of DCMU and dinoseb on cyt b_559_ photoreduction in the unsupplemented and PQ-supplemented PSII membranes

**Fig 4:**
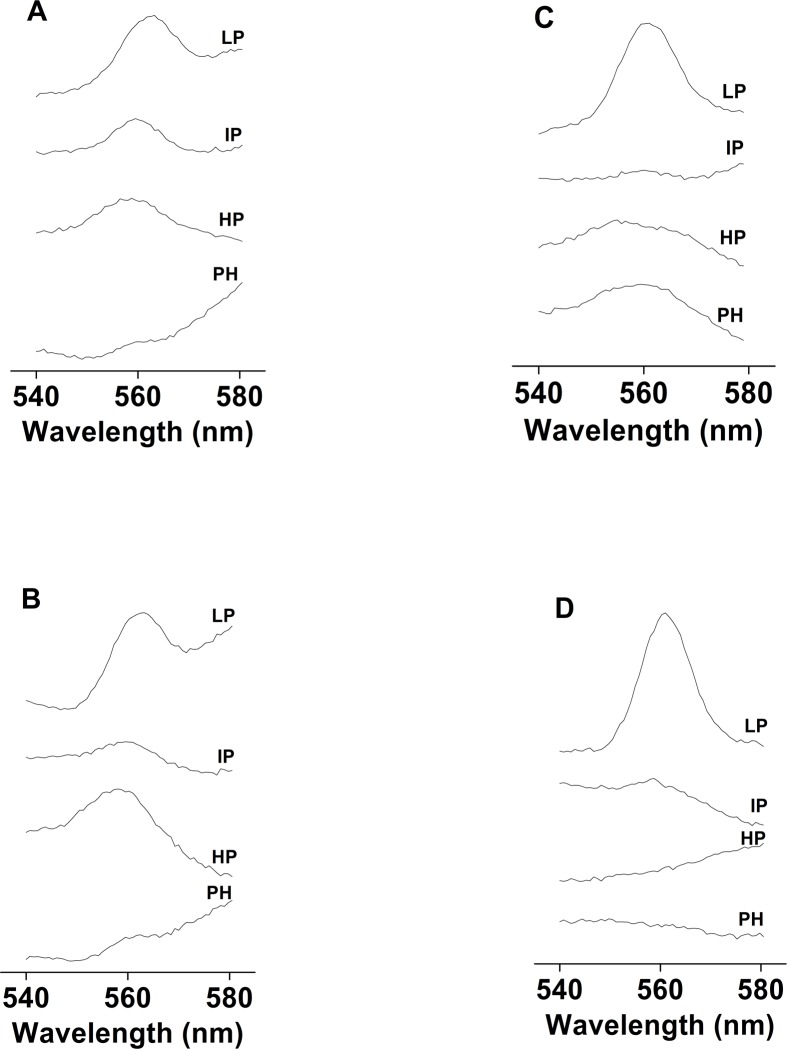
The effects of DCMU and dinoseb on cyt *b*_559_ photoreduction measured using unsupplemented and PQ-supplemented PSII membranes. Cyt *b*_559_ photoreduction was measured using unsupplemented [A,C] and PQ-supplemented [B,D] PSII membranes in the presence of DCMU [A, B] and dinoseb [C, D]. The other experimental conditions were the same as described in Fig. 3.

To confirm the involvement of the Q_B_ site in cyt *b*_559_ photoreduction via mobile plastoquinol, cyt *b*_559_ photoreduction was measured in the presence of DCMU. Addition of DCMU to unsupplemented or PQ-supplemented PSII membranes prior to illumination fully prevented photoreduction of the HP form of cyt *b*_559_ (Fig. 4A and 4B). These results indicate that DCMU prevents photoreduction of HP form of cyt *b*_559_ due to inhibition of plastoquinol formation. To confirm the involvement of the Q_D_ site in the photoreduction of cyt *b*_559_, cyt *b*_559_ photoreduction was measured in the presence of dinoseb. Illumination of PSII membranes in the presence of dinoseb did not cause cyt *b*_559_ photoreduction in both unsupplemented (Fig. 4C) and PQ-supplemented PSII membranes (Fig. 4D). These results suggest that dinoseb convert HP form to LP form of cyt *b*_559_ and prevents reduction of cyt *b*_559_ at the Q_D_ site due to inhibition of plastoquinol oxidation.

### 8. Quantifying loosely bound PQ and chlorophyll in PSII

To correlate the PQ-binding site and O_2_^•−^ formation in PSII membranes, the content of loosely bound plastoquinone was measured by HPLC. HPLC analysis of the chlorophyll content indicated approximately 250 chlorophyll molecules per reaction center (RC), consistent with values in the literature (i.e., 200–300 Chl/RC) [35, 42]. HPLC analysis of plastoquinone levels demonstrated that two of three plastoquinones per RC were extractable from the PSII membranes. These observations suggest that one plastoquinone is tightly bound (Q_A_) and two plastoquinones are loosely bound (Q_B_ and Q_C_ or Q_D_).

## Discussion

**Fig 5:**
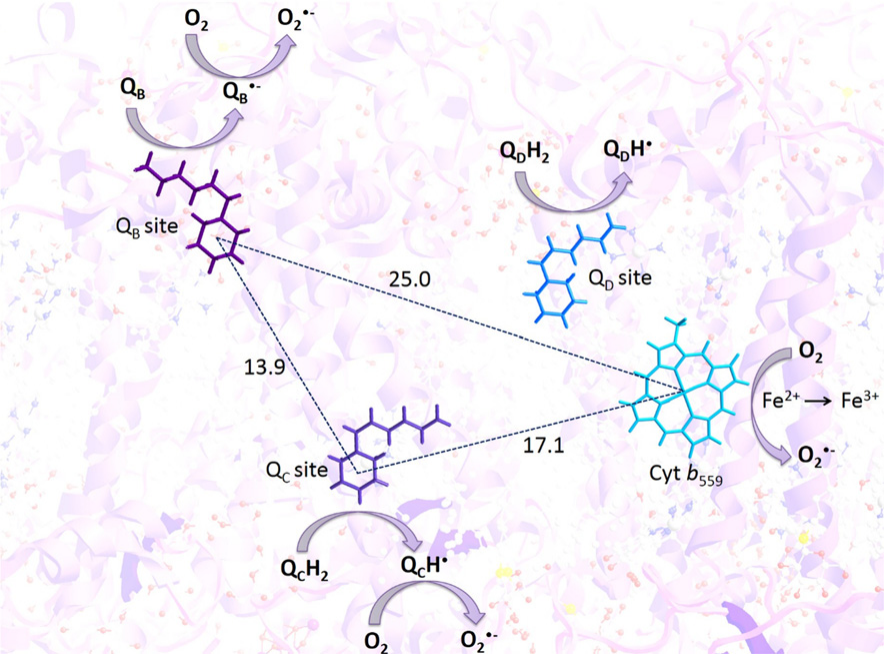
Proposed mechanism for the involvement of loosely bound plastosemiquinone at the Q_B_ and Q_C_ sites in O_2_^•–^ formation in PSII. Superoxide anion radicals are produced via one-electron reduction of molecular oxygen by plastosemiquinones, which are formed via one-electron reduction of plastoquinone at the Q_B_ sites and one-electron oxidation of plastoquinol at the Q_C_ site but unlikely at the Q_D_ site.

Several lines of evidence have been provided that O_2_^•−^ is formed through one-electron reduction of molecular oxygen on the electron acceptor side of PSII [16, 17]. As the operational redox potential for the O_2_/O_2_^•−^ redox couple is close to 0 mV or even positive due to the difference in concentration of molecular oxygen and O_2_^•−^, O_2_^•−^ formation requires a suitable electron donor with a redox potential lower than the operational redox potential of O_2_/O_2_^•−^ redox couple, and thus consequently, a high reducing power to reduce molecular oxygen. It was suggested that various cofactors on the electron acceptor side of PSII can fulfil such thermodynamic criteria and thus might serve as potential electron donors to molecular oxygen. Although light-induced O_2_^•−^ formation in PSII has been examined by measuring oxygen consumption [43–45], ferricytochrome c reduction and the xanthine/xanthine oxidase assay [22], voltametric methods [23] and EPR spin-trapping spectroscopy [20, 24, 26, 40–41, 46, 47], the molecular mechanism underlying light-induced O_2_^•−^ formation remains unclear. Here, we studied the role of loosely bound plastosemiquinone at the Q_B_, Q_C_ and Q_D_ sites in light-induced O_2_^•−^ formation in the PSII membranes supplemented with exogenous PQ-1. Addition of exogenous PQ-1 to the PSII membranes enhanced light-induced O_2_^•−^ production, indicating the involvement of plastosemiquinones in O_2_^•−^ production. Because the midpoint redox potentials for tightly bound plastosemiquinones at the Q_A_ site (*E*_m_ (Q_A_/Q_A_^•−^) = -60 to -140 mV, pH 7) [29–30] and loosely bound plastosemiquinone at the Q_B_ site (*E*_m_ (Q_B_/Q_B_^•−^) = -45 mV, pH 7) [31] are lower than the operational redox potential of O_2_/O_2_^•−^ redox couple (close to 0 mV or even positive), the reduction of molecular oxygen by plastosemiquinones is feasible. Based on the presented data, we propose that O_2_^•−^ is produced by one-electron reduction of molecular oxygen by plastosemiquinones formed by one-electron reduction of plastoquinone at the Q_B_ sites and one-electron oxidation of plastoquinol at the Q_C_ site but most likely not the Q_D_ site (Fig. 5).

### 1. Involvement of the Q_B_ site in O_2_^•−^ production

In the EPR spin-trapping data obtained using the urea-type herbicide DCMU, the EMPO-OOH adduct EPR signal was only partially suppressed, which indicates that molecular oxygen is reduced prior to the Q_B_ site (Fig. 2A and B). The DCMU-insensitive EMPO-OOH adduct EPR signal (50%) is likely due to reduction of molecular oxygen by Pheo^•−^ or Q_A_^•−^. It has been previously proposed that Pheo^•−^ and Q_A_^•−^ function as the predominant electron donors to molecular oxygen due to their low redox potentials [20, 22, 23, 48]. The DCMU-sensitive EMPO-OOH adduct EPR signal (50%) corresponds to the formation of O_2_^•−^ via reduction of molecular oxygen by plastosemiquinone formed at or after the Q_B_ site. Electron transfer from Q_A_^•−^ to loosely bound plastoquinone at the Q_B_ site yields Q_B_^•−^, which subsequently forms the more stable Q_B_H^•^ by protonation of proximal amino acids. Subsequent Q_B_H^•^ reduction and protonation yield Q_B_H_2_, which moves out through the channels [11]. However, if protonation of Q_B_^•−^ by proximal amino acids slows, the lifetime of Q_B_^•−^ increases. When molecular oxygen is in the proximity to Q_B_^•−^, reduction of molecular oxygen by Q_B_^•−^ produces O_2_^•−^.

### 2. Involvement of the Q_C_ site in O_2_^•−^ production

Based on X-ray crystal structural analyses of the PSII complex, Q_B_H_2_ exchange by plastoquinone at the Q_B_ site was proposed to occur via plastoquinol diffusion through channel I (bottom channel) and II (upper channel) [2]. During this process, Q_B_H_2_ liberates from the Q_B_ site and diffuses through the bottom channel to the Q_C_ site located in the vicinity of the heme iron of cyt *b*_559_ at distance of 17 Å from the head group of plastoquinol. Plastoquinol binding at the Q_C_ site was proposed to favour electron donation to the ferric heme iron of cyt *b*_559_ [46]. Illumination of PSII membranes caused the photoreduction of the HP form of cyt *b*_559_, demonstrating that Q_C_H_2_ is oxidized by the ferric heme iron of cyt *b*_559_ to form Q_C_H^•^. Here, we propose that Q_C_H^•^ reduces molecular oxygen to O_2_^•−^. Because addition of dinoseb to the PSII membranes partially enhanced O_2_^•−^ formation (Fig. 2C), we propose that the ferrous heme iron of LP cyt *b*_559_ reduces molecular oxygen, which forms O_2_^•−^. Fig. 4C and D) show that the HP form of cyt *b*_559_ was converted to the LP form in the presence of dinoseb, as previously demonstrated by Kaminskaya and Shuvalov [15]. In addition to binding of dinoseb to Q_D_ site which has been claimed in the recent past_,_ it is also known to bind to Q_B_ site. In such a case, the formation of Q_C_H^•^ is unlikely formed by oxidation of Q_c_H_2_; however, the alternative reaction pathway for formation of Q_C_H^•^ occurs. Consistent with this proposal, the formation of Q_C_H^•^ by one-electron reduction of plastoquinone cannot be excluded [26] and thus the involvement of Q_C_H^•^ and LP form of cyt *b*_559_ in O_2_^•−^ formation via the Q_C_ site might be considered.

### 3. Involvement of the Q_D_ site in O_2_^•−^ production

The observation that the phenolic-type herbicide dinoseb, which binds at the Q_D_ site enhanced EMPO-OOH adduct EPR signal further indicates that Q_D_H formed by plastoquinol oxidation at the Q_D_ site is not involved in O_2_^-^ production (Fig. 2A). Q_D_H_2_ oxidation by the heme iron of the HP form of cyt *b*_559_ and deprotonation by proximal amino acids results in the formation of Q_D_H^•^. Kaminskaya and Shuvalov [15] recently suggested that Q_D_H^•^ is stable at the Q_D_ site, and the midpoint redox potentials of the Q_D_/Q_D_H^•^ redox couple are more positive than those of the Q_B_/Q_B_^•−^ redox couple (*E*m = -45 mV, pH 7). Consistent with this proposal, we assume that the reduction of molecular oxygen by Q_D_H^•^ is not feasible and thus O_2_^•−^ formation at the Q_D_ site is ambiguous.

## Conclusion

The data presented in this study demonstrate that loosely bound plastosemiquinones at the Q_B_ and Q_C_ sites are involved in the formation of O_2_^•−^ via one-electron reduction of molecular oxygen. Loosely bound plastosemiquinone Q_B_^•−^ is formed via one-electron reduction of plastoquinone at the Q_B_ site; however, one-electron oxidation of plastoquinol by cyt *b*_559_ at the Q_C_ site forms Q_C_H^•^. By contrast, the results indicated that O_2_^•−^ formation from plastosemiquinones at the Q_D_ site was ambiguous. In addition to loosely bound plastosemiquinone, previous studies have reported the formation of O_2_^•-^ by free plastosemiquinone in the PQ pool [25, 43–45]. The interaction of plastoquinol with plastoquinone in the PQ pool was suggested to result in the formation of free PQ^•−^, which reduces molecular oxygen to form O_2_^•−^. Further studies are needed to elucidate a unifying mechanism for O_2_^•-^ formation which involves PQ pool.
