# Correction: Evidence for the Involvement of Loosely Bound Plastosemiquinones in Superoxide Anion Radical Production in Photosystem II

**DOI:** 10.1371/journal.pone.0130244

**Published:** 2015-06-04

**Authors:** The PLOS ONE Staff

## Notice of Republication

This article was republished on May 13, 2015, because radical signs used to differentiate native and radical forms of species were omitted throughout the paper. These errors were introduced during the typesetting process. The publisher apologizes for these errors. The originally published, uncorrected article and the republished, corrected article are provided here for reference.

## Supporting Information

S1 File Originally published, uncorrected article. (PDF)

S2 File Republished corrected article. (PDF)
