# Supplementary material for: Correction: Evidence for the Involvement of Loosely Bound Plastosemiquinones in Superoxide Anion Radical Production in Photosystem II
Source: PLoS One. 2015 Jun 4;10(6):e0130244. doi: 10.1371/journal.pone.0130244 (PMC4456419; doi:10.1371/journal.pone.0130244)
Supplement: S1 File — (PDF) [file pone.0130244.s001.pdf]

PLOS ONE

RESEARCH ARTICLE

Evidence for the Involvement of Loosely Bound Plastosemiquinones in Superoxide Anion Radical Production in Photosystem II

Deepak Kumar Yadav[1], Ankush Prasad[1], Jerzy Kruk[2], Pavel Pospíšil[1]*

1. Department of Biophysics, Centre of the Region Haná for Biotechnological and Agricultural Research, Faculty of Science, Palacký University, Olomouc, Czech Republic, **2.** Department of Plant Physiology and Biochemistry, Faculty of Biochemistry, Biophysics and Biotechnology, Jagiellonian University, Kraków, Poland

*pavel.pospisil@upol.cz

CrossMark
click for updates

OPEN ACCESS

Citation: Yadav DK, Prasad A, Kruk J, Pospíšil P (2014) Evidence for the Involvement of Loosely Bound Plastosemiquinones in Superoxide Anion Radical Production in Photosystem II. PLoS ONE 9(12): e115466. doi:10.1371/journal.pone.0115466

Editor: Maya Dimova Lambreva, National Research Council of Italy, Italy

Received: September 5, 2014

Accepted: November 24, 2014

Published: December 26, 2014

Copyright: © 2014 Yadav et al. This is an open-access article distributed under the terms of the Creative Commons Attribution License, which permits unrestricted use, distribution, and reproduction in any medium, provided the original author and source are credited.

Data Availability: The authors confirm that all data underlying the findings are fully available without restriction. All relevant data are within the paper.

Funding: This work was supported by the Ministry of Education, Youth and Sports of the Czech Republic grants no. LO1204 (National Program of Sustainability I), no. CZ.1.07/2.3.00/20.0057 (Progress and Internationalization of Biophysical Research at the Faculty of Science, Palacký University) and no. CZ.1.07/2.3.00/30.0041 (Support for Building Excellent Research Teams and Intersectoral Mobility at Palacký University). The funders had no role in study design, data collection and analysis, decision to publish, or preparation of the manuscript.

Competing Interests: The authors have declared that no competing interests exist.

Abstract

Recent evidence has indicated the presence of novel plastoquinone-binding sites, Q_C and Q_D, in photosystem II (PSII). Here, we investigated the potential involvement of loosely bound plastosemiquinones in superoxide anion radical (O_2^{-}) formation in spinach PSII membranes using electron paramagnetic resonance (EPR) spin-trapping spectroscopy. Illumination of PSII membranes in the presence of the spin trap EMPO (5-(ethoxycarbonyl)-5-methyl-1-pyrroline N-oxide) resulted in the formation of O_2^{-}, which was monitored by the appearance of EMPO-OOH adduct EPR signal. Addition of exogenous short-chain plastoquinone to PSII membranes markedly enhanced the EMPO-OOH adduct EPR signal. Both in the unsupplemented and plastoquinone-supplemented PSII membranes, the EMPO-OOH adduct EPR signal was suppressed by 50% when the urea-type herbicide DCMU (3-(3,4-dichlorophenyl)-1,1-dimethylurea) was bound at the Q_B site. However, the EMPO-OOH adduct EPR signal was enhanced by binding of the phenolic-type herbicide dinoseb (2,4-dinitro-6-sec-butylphenol) at the Q_D site. Both in the unsupplemented and plastoquinone-supplemented PSII membranes, DCMU and dinoseb inhibited photoreduction of the high-potential form of cytochrome b_{559} (cyt b_{559}). Based on these results, we propose that O_2^{-} is formed via the reduction of molecular oxygen by plastosemiquinones formed through one-electron reduction of plastoquinone at the Q_B site and one-electron oxidation of plastoquinol by cyt b_{559} at the Q_C site. On the contrary, the involvement of a plastosemiquinone formed via the one-electron oxidation of plastoquinol by cyt b_{559} at the Q_D site seems to be ambiguous. In spite of the fact that the existence of Q_C and Q_D sites is not generally

accepted yet, the present study provided more spectroscopic data on the potential functional role of these new plastoquinone-binding sites.

Introduction

Photosystem (PSII) is a heterodimeric multiprotein-pigment complex embedded in the thylakoid membrane of photosynthetic organisms such as cyanobacteria, algae and higher plants. Recent X-ray crystallographic structural analyses of PSII from the cyanobacteria *Thermosynechococcus elongatus* and *Thermosynechococcus vulcanus* demonstrated that PSII consists of 20 protein subunits, 35 chlorophylls, 12 carotenoids and 25 lipids per monomer [1–3]. During oxygenic photosynthesis, PSII functions as a water-plastoquinone oxidoreductase that oxidizes water to molecular oxygen and reduces plastoquinone to plastoquinol [4–5]. In these reactions, four electrons extracted from water by a water-splitting manganese complex on the electron donor side of PSII are transferred to the primary and secondary electron acceptors on the electron acceptor side of PSII [6–9]. It is well established that the primary and secondary electron acceptors are plastoquinones tightly and loosely bound to the Q_A and Q_B sites, respectively. One-electron reduction of plastoquinone at the Q_B site forms plastosemiquinone (Q_B^{-}), which is subsequently stabilized by the protonation of proximal amino acid side chains (Q_BH), whereas the sequential one-electron reduction and protonation of Q_BH forms plastoquinol (Q_BH_2).

Several biochemical studies have suggested that PSII contains two plastoquinone-binding sites in addition to the Q_A and Q_B sites [10–12]. Based on the study on photoreduction of cytochrome b_{559} (cyt b_{559}) in the presence of exogenous plastoquinone, a third plastoquinone -binding site referred to as Q_C was proposed to be located closed to cyt b_{559} [10]. Later, the effects of herbicides and ADRY agents on the redox properties of cyt b_{559} provided more biochemical data on the existence of Q_C site [11–12]. Consistent with biochemical studies, the crystal structure of PSII at 2.9 Å resolution revealed the existence of Q_C site [2]. However, the Q_C site was not reported in the most recent PSII crystal structure at 1.9 Å resolution [3]. Hasegawa and Noguchi proposed that the affinity of plastoquinone to the Q_C site is lower compared to the Q_B site [13]. In agreement with this proposal, it has been recently suggested that ambiguity in the existence of Q_C site might be due to the different purification and crystallization procedures [14]. Recently, Kaminskaya and Shuvalov [15] identified a fourth plastoquinone-binding site denoted as Q_D. The authors concluded that the Q_C site depicted in the PSII crystal structure is in a highly hydrophobic environment, while the Q_D site is located in a more polar environment. The urea-type herbicide DCMU (3-(3,4-dichlorophenyl)-1,1-dimethylurea) blocks Q_B to Q_B^{-} reduction at the Q_B site, whereas the phenolic-type herbicide dinoseb (2,4-dinitro-6-sec-butylphenol)

prevents the oxidation of plastoquinol (Q_DH_2) to plastosemiquinone (Q_DH) by cyt b_{559} at the Q_D site [15].

The limitations on electron transport both on the electron donor and acceptor sides of PSII are associated with the formation of reactive oxygen species (ROS) [16–19]. Under high-light conditions, when light absorption by chlorophylls exceeds the utilization of excitation energy, the over-reduction of the electron acceptor side of PSII leads to leakage of electrons to molecular oxygen. The reduction of molecular oxygen results in the formation of superoxide anion radical (O_2^{-}), which either spontaneously dismutates to hydrogen peroxide (H_2O_2) or forms bound peroxide through interactions with the non-heme [20] or heme iron in cyt b_{559} [21]. Subsequent reductions of either H_2O_2 by free metals or bound peroxide by the non-heme iron forms hydroxyl radicals (HO) [20].

Several studies have demonstrated that various cofactors on the electron acceptor side of PSII can reduce molecular oxygen, forming O_2^{-}. These cofactors are highly reducing species with a midpoint redox potential lower than the standard redox potential of the O_2/O_2^{-} redox couple ($E_0'=-160$ mV, pH 7). Molecular oxygen may be reduced by pheophytin ($Pheo^{-}$) [20, 22], the tightly bound plastosemiquinone at the Q_A site (Q_A^{-}) [23], the loosely bound plastosemiquinone at the Q_B site (Q_B^{-}) [24], free plastosemiquinone (PQ^{-}) [25] and the ferrous heme iron in the low-potential (LP) form of cyt b_{559} [26].

Due to a highly negative redox potential (Em ($Pheo/Pheo^{-}$) $=-505$ to -610 mV, pH 6.5 to 7) [27–28], the reduction of molecular oxygen by $Pheo^{-}$ is likely. The favorable thermodynamic properties for reduction of molecular oxygen by $Pheo^{-}$ are limited by kinetic restrictions. Forward electron transport from $Pheo^{-}$ to Q_A^{-} is much more rapid than diffusion-limited reduction of molecular oxygen; thus, the reduction of molecular oxygen by $Pheo^{-}$ is less likely. However, under certain circumstances, such as limitation of electron transport from $Pheo^{-}$ to Q_A^{-}, the $Pheo^{-}$ lifetime is prolonged, and the reduction of molecular oxygen is more likely.

In contrast to $Pheo^{-}$, reduction of molecular oxygen by Q_A^{-} and Q_B^{-} is less favorable from a thermodynamic perspective. In principle, the midpoint redox potentials of the Q_A/Q_A^{-} (Em $=-60$ to -140 mV, pH 7) [29–30] and Q_B/Q_B^{-} (Em$=-45$ mV, pH 7) [31] redox couples are greater than the standard redox potential of the O_2/O_2^{-} redox couple ($E_0'=-160$ mV, pH 7) [32]. When the concentrations of reactant (O_2 ~ hundreds µM) and product (O_2^{-} ~ hundreds nM) differ, the operational redox potential of the O_2/O_2^{-} redox couple is shifted to 0 mV or even positive values based on the Nernst equation [16–17]. Thus, the reduction of molecular oxygen by Q_A^{-} and Q_B^{-} seems to be more thermodynamically feasible. From a kinetic perspective, the lifetimes of Q_A^{-} and Q_B^{-} are sufficiently long for the diffusion-limited reduction of molecular oxygen. In addition to Q_A^{-} and Q_B^{-}, free PQ^{-} can reduce molecular oxygen (Em$=-170$ mV, pH 7) [31]; however, the probability of its formation by the interaction of free plastoquinone and free plastoquinol is very low [25]. It has been proposed that the reduction of molecular oxygen by ferrous heme iron in the LP form of cyt b_{559} produces O_2^{-} and may be thermodynamically feasible because

the LP form of cyt b_{559} has a low midpoint redox potential (Em = −40 to +80 mV, pH 7) [21, 26, 33].

Herein, we studied whether loosely bound plastosemiquinones are involved in the light-induced O_2^{-} formation in PSII membranes using an electron paramagnetic resonance (EPR) spin-trapping spectroscopy. We provide evidence that O_2^{-} is produced via one-electron reduction of molecular oxygen by plastosemiquinones, which are formed through one-electron reduction of plastoquinone at the Q_B site (Q_B^{-}) and one-electron oxidation of plastoquinol by cyt b_{559} at the Q_C site (Q_CH). By contrast, a role of plastosemiquinone formed at the Q_D site (Q_DH) in O_2^{-} formation is ambiguous.

Materials and Methods

1. PSII membrane preparation

PSII membranes were isolated from fresh spinach leaves using the method reported previously by Berthold *et al.* [34] with modifications described by Ford and Evans [35]. The isolated PSII membranes were dissolved in a buffer solution containing 400 mM sucrose, 10 mM NaCl, 5 mM $CaCl_2$, 5 mM $MgCl_2$ and 50 mM Mes-NaOH (pH 6.5) and stored at −80℃ until further use. For PQ-supplemented PSII membranes, exogenous short-chain platoquinone containing one isoprenoid units in the side-chain (PQ-1) was added to the PSII membranes prior to illumination. 30 μM PQ-1 was added to the PSII membranes as an ethanol solution (the final concentration of ethanol did not exceed 1%).

2. EPR spin-trapping spectroscopy

O_2^{-} was detected by EPR spin-trapping spectroscopy using EMPO (5-(ethoxycarbonyl)-5-methyl-1-pyrroline N-oxide; Alexis Biochemicals, Lausen, Switzerland) as the spin trap. PSII membranes (150 μg Chl ml^{-1}) were illuminated with a continuous white light (1000 μmol photons m^{-2} s^{-1}) in a glass capillary tube (Blaubrand intraMARK, Brand, Germany) with 25 mM EMPO, 100 μM Desferal and 40 mM MES buffer (pH 6.5). PSII membranes were illuminated using a halogen lamp with a light guide (Schott KL 1500, Schott AG, Mainz, Germany) at room temperature. The spectra were recorded using an EPR spectrometer Mini Scope MS400 (Magnettech GmbH, Germany). The following EPR conditions were used: microwave power, 10 mW; modulation amplitude, 1 G; modulation frequency, 100 kHz; sweep width, 100 G; and scan rate, 1.62 G s^{-1}. For quantification, intensity of EPR signal was evaluated as the relative height of peak of the first derivative of the EPR absorption spectrum.

3. Optical measurements

The redox properties of cyt b_{559} were studied using an Olis RSM 1000 spectrometer (Olis Inc., Bogart, Georgia, USA). The redox states of cyt b_{559} in PSII membranes (150 μg Chl ml^{-1}) were determined based on the changes in the

absorbance at 559 nm upon stepwise additions of 50 μM potassium ferricyanide, 8 mM hydroquinone, 5 mM sodium ascorbate and sodium dithionite in a cuvette at room temperature using the method in Tiwari and Pospíšil [21] with certain modification. The redox forms of cyt b_{559} in the PSII membranes were determined by subtracting the control from the treatment spectra: for the HP form of cyt b_{559}, the hydroquinone-reduced spectra were subtracted from the ferricyanide-oxidized cyt b_{559}; for the IP form of cyt b_{559}, the ascorbate-reduced spectra were subtracted from the hydroquinone-reduced cyt b_{559}; and for the LP form of cyt b_{559}, the dithionite-reduced spectra were subtracted from the ascorbate-reduced cyt b_{559}. In photoreduction measurements, the photoreduced HP form of cyt b_{559} (PH) was calculated based on the difference between the absorbance spectra measured after illumination for 180 s and the dark-adapted ferricyanide oxidized spectrum and hydroquinone-reduced spectra were subtracted from photoreduced HP form of cyt b_{559} to get unreduced HP form of cyt b_{559}. The PSII membranes were illuminated with continuous white light (1000 μmol photons m^{-2} s^{-1}) in the cuvette, which was rotated by 90° at intervals of 15 s.

4. High-pressure liquid chromatography

The loosely bound plastoquinone was measured using the method in Wydrzynski and Inoue [36]. A 1 ml aliquot of the PSII membranes (300 μg Chl ml^{-1}) was mixed with 3 ml of heptane and 30 μl of isobutanol, followed by vortexing for 1 h in the dark. The mixture was then centrifuged at 4000 × *g* for 10 min. The plastoquinone content in the upper organic layer was determined by HPLC based on the method of Kruk and Karpinski [37].

Results

1. Superoxide anion radical production in unsupplemented PSII membranes

Light-induced O_2^{-} formation in the unsupplemented PSII membranes was measured using EPR spin-trapping spectroscopy. For spin-trapping, we used the spin trap compound EMPO, which reacts with O_2^{-} to form an EMPO-OOH adduct [38]. No EMPO-OOH adduct EPR signal appeared immediately after addition of EMPO to the unsupplemented PSII membranes in the dark (Fig. 1A). Illumination of the unsupplemented PSII membranes in the presence of EMPO resulted in the production of an EMPO-OOH adduct EPR signal (Fig. 1A). To prevent EMPO-OH adduct formation, the strong iron chelator Desferal was used to decrease the level of free iron available to produce HO through the Fenton reaction [26, 39]. Fig. 2B shows the time profile for the EMPO-OOH adduct EPR signal measured for the unsupplemented PSII membranes. These results demonstrate that the illumination of unsupplemented PSII membranes results in the formation of O_2^{-}.

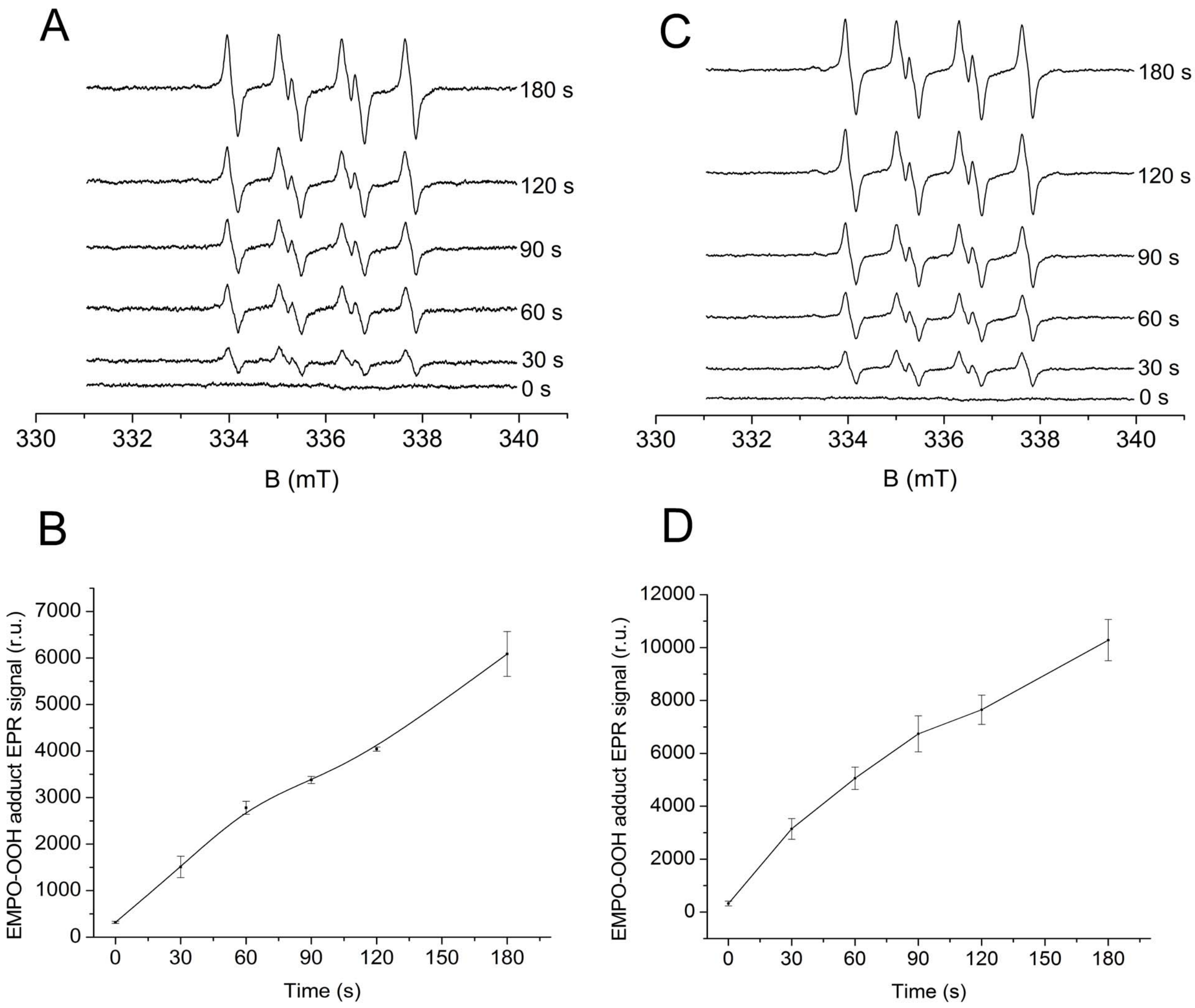

Fig. 1. Light-induced EMPO-OOH adduct EPR spectra measured using unsupplemented and PQ-supplemented PSII membranes. EMPO-OOH adduct EPR spectra were obtained after illumination of PSII membranes (150 μg Chl ml^{-1}) with white light (1000 μmol photons m^{-2} s^{-1}) in the absence [A, B] and presence of exogenous PQ-1 [C, D] and in the presence of 25 mM EMPO, 100 μM Desferal and 40 mM MES (pH 6.5). Figures B and D shows mean ± SD, where n=3. 30 μM PQ-1 was added to PSII membranes prior to illumination.

doi:10.1371/journal.pone.0115466.g001

2. Superoxide anion radical production in PQ-supplemented PSII membranes

To study the role of loosely bound plastosemiquinone in O_2^{-} formation, light-induced O_2^{-} formation was measured in the presence of exogenous PQ-1. Because PQ-1 is smaller than the natural molecule PQ-9, PQ-1 can better penetrate the membrane and substitute for PQ-9 as an electron acceptor in PSII. The observation that the addition of PQ-1 to EMPO did not generate any EPMO-OOH adduct EPR spectrum indicates that PQ-1 does not directly interact with EMPO (data not shown). In the dark, the addition of PQ-1 to the PSII membranes in the presence of EMPO did not produce an EPR signal; however, exposure of PQ-supplemented PSII membranes to white light resulted in the

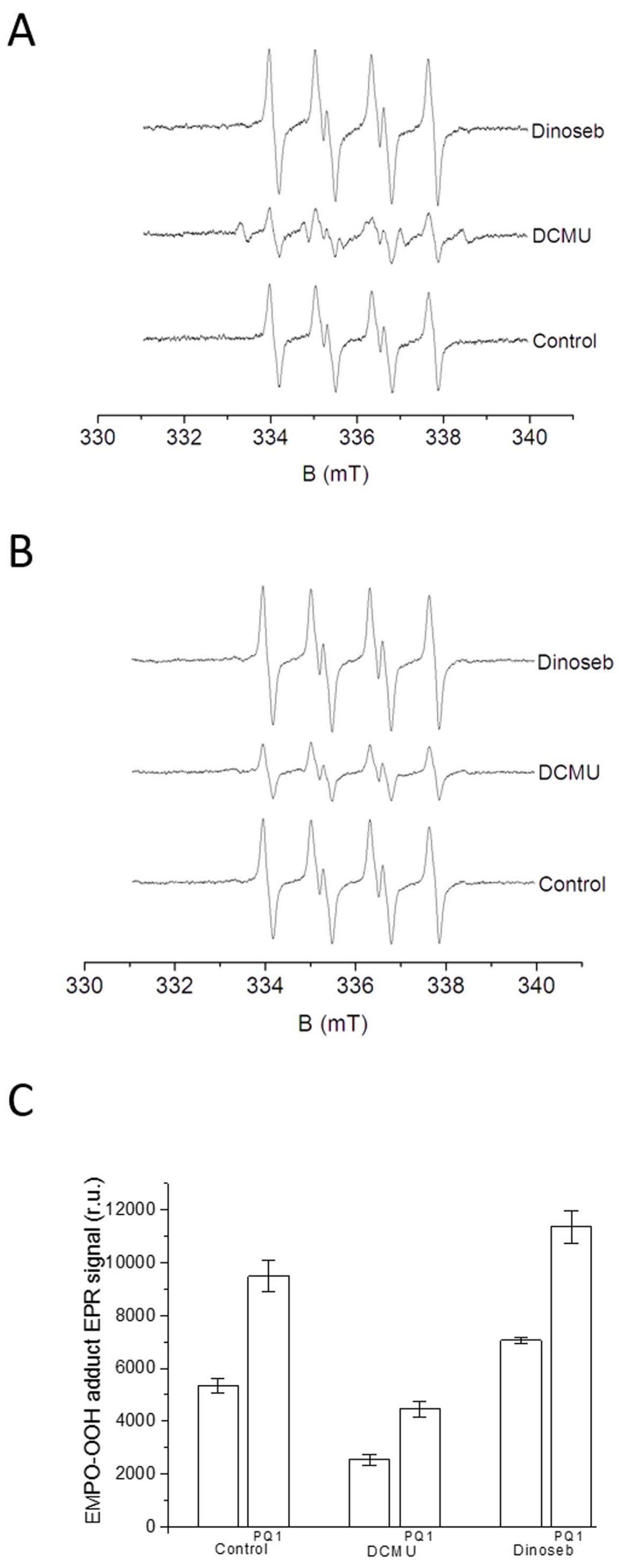
A
Dinoseb
DCMU
Control
330
332
334
336
338
340
B (mT)
B
Dinoseb
DCMU
Control
330
332
334
336
338
340
B (mT)
C
EMPO-OOH adduct EPR signal (r.u.)
0
2000
4000
6000
8000
10000
12000
PQ1
Control
PQ1
DCMU
PQ1
Dinoseb

Fig. 2. The effects of DCMU and dinoseb on EMPO-OOH adduct EPR spectra measured using unsupplemented and PQ-supplemented PSII membranes. EMPO-OOH adduct EPR spectra were measured using unsupplemented [A] and PQ-supplemented PSII membranes [B] in the presence of DCMU and dinoseb. Prior to illumination, DCMU (20 μM) and dinoseb (200 μM) were added to the membranes. [C] The relative intensity (mean ± SD, n=3) of the light-induced EMPO-OOH adduct EPR signal measured using unsupplemented and PQ-supplemented PSII membranes. The other experimental conditions were the same as described in Fig. 1.

doi:10.1371/journal.pone.0115466.g002

formation of an EMPO-OOH adduct EPR signal (Fig. 1C). The time profile of the EMPO-OOH adduct EPR signal measured after addition of exogenous PQ-1 to the PSII membranes revealed that the intensity of the EMPO-OOH adduct EPR signal was enhanced by 70% as compared to unsupplemented PSII membranes (Fig. 1D). These results indicate that plastosemiquinones are involved in light-induced O_2^- production in PSII.

3. The effects of DCMU and dinoseb on superoxide anion radical production in unsupplemented PSII membranes

To investigate where loosely bound plastosemiquinones involved in O_2^- production are formed, the effects of two herbicides, DCMU (bound at the Q_B site) and dinoseb (bound at the Q_D site) on the EMPO-OOH adduct EPR signal were studied in the unsupplemented PSII membranes. When the unsupplemented PSII membranes were illuminated in the presence of DCMU, the EMPO-OOH adduct EPR signal was suppressed by 50%, whereas the remaining EPMO-OOH EPR signal (50%) was insensitive to DCMU (Fig. 2A and C). In previous studies [24, 26, 40, 41], the relative proportion of DCMU-sensitive and DCMU-insensitive O_2^- production in PSII varied, likely due to the endogenous platoquinone content. In addition to the EMPO-OOH adduct EPR signal, the EPR spectrum measured in the presence of DCMU comprises an EMPO-R adduct EPR signal formed by the interaction between EMPO and a carbon-centered radical, the origin of which is unknown. These observations reveal that 1) the DCMU-sensitive EMPO-OOH adduct EPR signal corresponds to O_2^- formed at or after the Q_B site (i.e., reduction of molecular oxygen by loosely bound plastosemiquinones formed by one-electron reduction of plastoquinone and one-electron oxidation of plastoquinol) and 2) the DCMU-insensitive EMPO-OOH adduct EPR signal corresponds to O_2^-, which is formed before the Q_B site (i.e., reduction of molecular oxygen by $Pheo^-$ and Q_A^-). When dinoseb was added to the unsupplemented PSII membranes prior to illumination, the EMPO-OOH adduct EPR signal was enhanced by 25% (Fig. 2A and C). Due to the fact that the occupation of the Q_D site does not eliminate O_2^- production, the production of O_2^- by reduction of molecular oxygen by plastosemiquinone at the Q_D site is ambiguous.

4. The effects of DCMU and dinoseb on superoxide anion radical production in PQ-supplemented PSII membranes

Addition of DCMU to PQ-supplemented PSII membranes decreased the EMPO-OOH adduct EPR signal by 55% (Fig. 2B and C). Similar to unsupplemented PSII membranes, in PQ-supplemented PSII membranes, 1) O_2^{-} is formed at or after the Q_B site via reduction of molecular oxygen by plastosemiquinone formed via one-electron reduction of plastoquinone and one-electron oxidation of plastoquinone and 2) O_2^{-} is formed prior to the Q_B site by reduction of molecular oxygen by $Pheo^{-}$ and Q_A^{-}. The intensity of the EMPO-OOH adduct EPR signal after the addition of DCMU was higher for the PQ-supplemented PSII membranes than for the unsupplemented PSII membranes (Fig. 2C). When dinoseb was added to the PQ-supplemented PSII membranes prior to illumination, the EMPO-OOH adduct EPR signal was enhanced by 17% (Fig. 2B and C). The intensity of the EMPO-OOH adduct EPR signal after the addition of dinoseb was higher for PQ-supplemented PSII membranes compared to unsupplemented PSII membranes (Fig. 2C). Similar to the unsupplemented PSII membranes, the effect of dinoseb on O_2^{-} production in PQ-supplemented PSII membranes indicate that the Q_D site is unlikely involved in O_2^{-} production.

5. Different redox forms of cyt b_{559} in the unsupplemented and PQ-supplemented PSII membranes

To determine the different redox forms of cyt b_{559}, we measured changes in absorption at 559 nm in the unsupplemented and PQ-supplemented PSII membranes. The different redox forms of cyt b_{559} were discerned by examining the hydroquinone-reduced minus ferricyanide-oxidized (HP) spectra, ascorbate-reduced minus hydroquinone-reduced (IP) spectra, and dithionite-reduced minus ascorbate-reduced (LP) spectra. In the unsupplemented PSII membranes, 40% of cyt b_{559} was in the hydroquinone-reducible HP form, 22% was in the sodium ascorbate-reducible IP form, and 38% was in the dithionite-reducible LP form (Fig. 3A). In the supplemented PSII membranes, the levels of the hydroquinone-reducible HP, sodium ascorbate-reducible IP and dithionite-reducible LP forms of cyt b_{559} were 42, 12 and 46% (Fig. 3B). These observations confirm the presence of the HP, IP and LP forms of cyt b_{559} in both unsupplemented and PQ-supplemented PSII membranes.

6. Cyt b_{559} photoreduction in the unsupplemented and PQ-supplemented PSII membranes

To observe the light-induced reducible redox form of cyt b_{559}, cyt b_{559} photoreduction was measured in both unsupplemented and PQ-supplemented PSII membranes. When the unsupplemented PSII membranes were exposed to white light, the HP form of cyt b_{559} was reduced (Fig. 3C). Addition of hydroquinone to the unsupplemented PSII membranes after illumination did not further reduce the HP form of cyt b_{559}; however, addition of sodium ascorbate

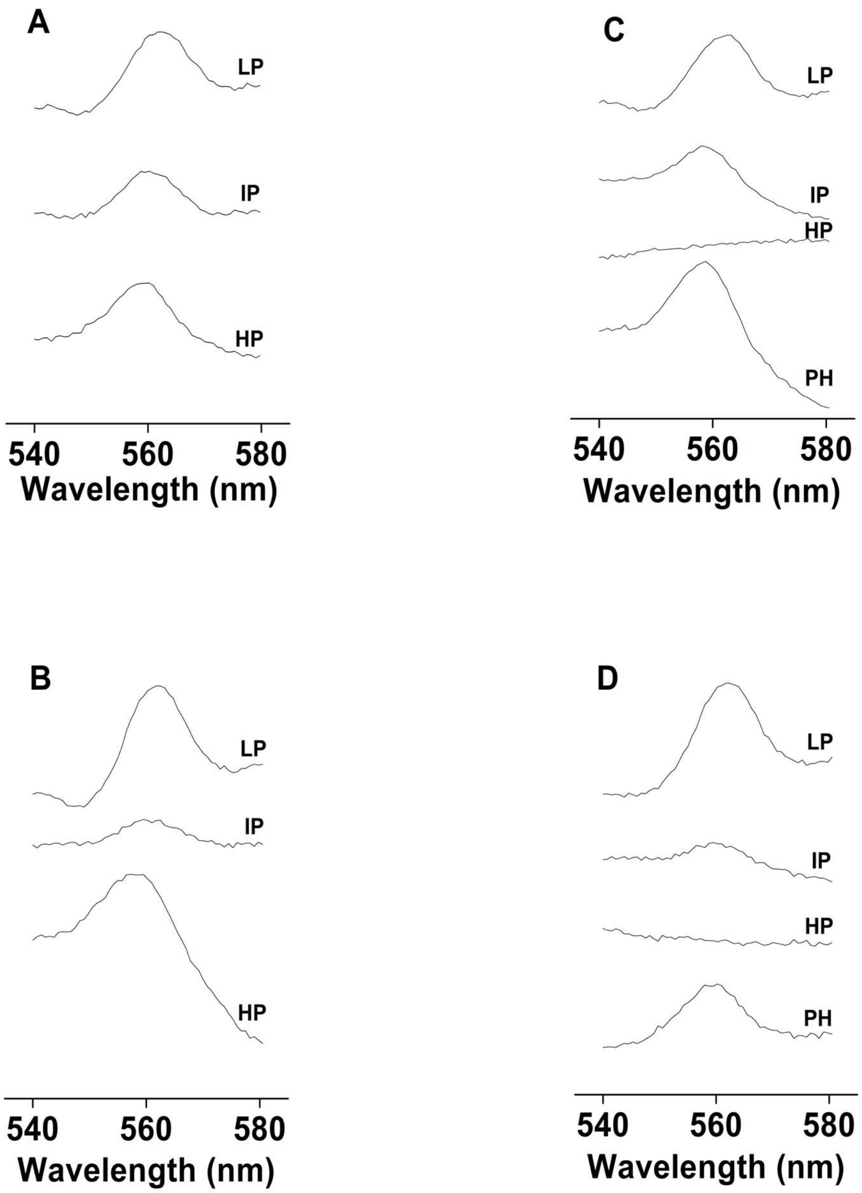

Fig. 3. Differences in redox spectra and cyt b_{559} photoreduction measured using unsupplemented and PQ-supplemented PSII membranes. Differences in the redox spectra of cyt b_{559} measured in the dark using unsupplemented [A] and PQ-supplemented PSII membranes [B]. 100 μM PQ-1 was added to the PSII membranes prior to the experiments. To measure cyt b_{559} photoreduction, unsupplemented [C] and PQ-supplemented PSII membranes [D] were illuminated for 180 s at high light intensity (1000 μmol photons m^{-2} s^{-1}). The spectra represent the difference in the light minus ferricyanide-oxidized spectra [the photoreduced HP form of cyt b_{559}, (PH)], hydroquinone-reduced minus ferricyanide-oxidized or hydroquinone-reduced minus light spectra [HP form of cyt b_{559}, (HP)], ascorbate-reduced minus hydroquinone-reduced spectra [IP form of cyt b_{559}, (IP)] and dithionite-reduced minus ascorbate-reduced spectra [LP form of cyt b_{559}, (LP)].

doi:10.1371/journal.pone.0115466.g003

and sodium dithionite reduced the IP and LP forms of cyt b_{559} (Fig. 3C). Similarly, exposure of PQ-supplemented PSII membranes to white light reduced the HP form of cyt b_{559} (Fig. 3D); however, addition of hydroquinone to

PQ-supplemented PSII membranes after illumination did not further reduce the HP form. Addition of ascorbate and dithionite to PQ-supplemented PSII membranes reduced the IP and LP forms of cyt b_{559} (Fig. 3D). These results demonstrate that illumination of the unsupplemented and PQ-supplemented PSII membranes reduced the HP form of cyt b_{559}.

7. The effects of DCMU and dinoseb on cyt b_{559} photoreduction in the unsupplemented and PQ-supplemented PSII membranes

To confirm the involvement of the Q_B site in cyt b_{559} photoreduction via mobile plastoquinol, cyt b_{559} photoreduction was measured in the presence of DCMU. Addition of DCMU to unsupplemented or PQ-supplemented PSII membranes prior to illumination fully prevented photoreduction of the HP form of cyt b_{559} (Fig. 4A and 4B). These results indicate that DCMU prevents photoreduction of HP form of cyt b_{559} due to inhibition of plastoquinol formation. To confirm the involvement of the Q_D site in the photoreduction of cyt b_{559}, cyt b_{559} photoreduction was measured in the presence of dinoseb. Illumination of PSII membranes in the presence of dinoseb did not cause cyt b_{559} photoreduction in both unsupplemented (Fig. 4C) and PQ-supplemented PSII membranes (Fig. 4D). These results suggest that dinoseb convert HP form to LP form of cyt b_{559} and prevents reduction of cyt b_{559} at the Q_D site due to inhibition of plastoquinol oxidation.

8. Quantifying loosely bound PQ and chlorophyll in PSII

To correlate the PQ-binding site and O_2^{-} formation in PSII membranes, the content of loosely bound plastoquinone was measured by HPLC. HPLC analysis of the chlorophyll content indicated approximately 250 chlorophyll molecules per reaction center (RC), consistent with values in the literature (i.e., 200–300 Chl/RC) [35, 42]. HPLC analysis of plastoquinone levels demonstrated that two of three plastoquinones per RC were extractable from the PSII membranes. These observations suggest that one plastoquinone is tightly bound (Q_A) and two plastoquinones are loosely bound (Q_B and Q_C or Q_D).

Discussion

Several lines of evidence have been provided that O_2^{-} is formed through one-electron reduction of molecular oxygen on the electron acceptor side of PSII [16, 17]. As the operational redox potential for the O_2/O_2^{-} redox couple is close to 0 mV or even positive due to the difference in concentration of molecular oxygen and O_2^{-}, O_2^{-} formation requires a suitable electron donor with a redox potential lower than the operational redox potential of O_2/O_2^{-} redox couple, and thus consequently, a high reducing power to reduce molecular oxygen. It was suggested that various cofactors on the electron acceptor side of PSII can fulfil such thermodynamic criteria and thus might serve as potential electron donors to

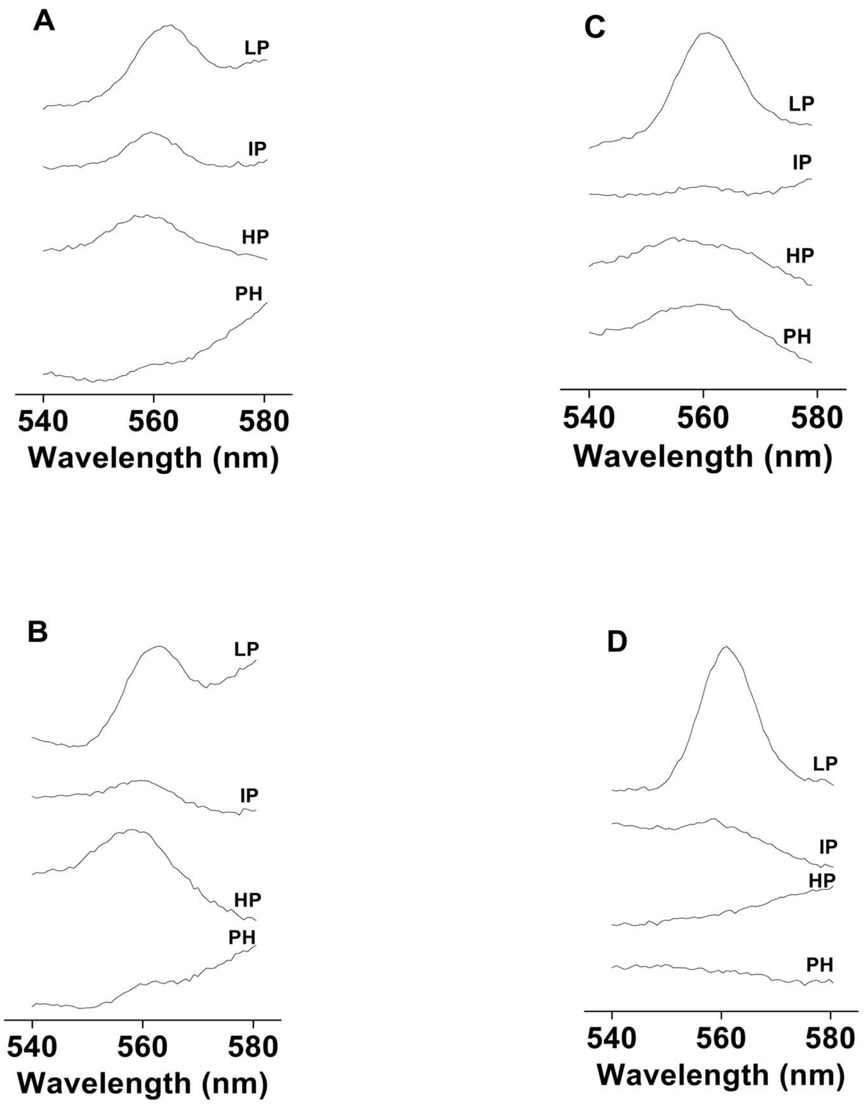

Fig. 4. The effects of DCMU and dinoseb on cyt b_{559} photoreduction measured using unsupplemented and PQ-supplemented PSII membranes. Cyt b_{559} photoreduction was measured using unsupplemented [A, C] and PQ-supplemented [B, D] PSII membranes in the presence of DCMU [A, B] and dinoseb [C, D]. The other experimental conditions were the same as described in Fig. 3.

doi:10.1371/journal.pone.0115466.g004

molecular oxygen. Although light-induced O_2^{-} formation in PSII has been examined by measuring oxygen consumption [43–45], ferricytochrome c reduction and the xanthine/xanthine oxidase assay [22], voltametric methods [23] and EPR spin-trapping spectroscopy [20, 24, 26, 40–41, 46, 47], the molecular mechanism underlying light-induced O_2^{-} formation remains unclear. Here, we studied the role of loosely bound plastosemiquinone at the Q_B, Q_C and Q_D sites in light-induced O_2^{-} formation in the PSII membranes supplemented with exogenous PQ-1. Addition of exogenous PQ-1 to the PSII membranes enhanced light-induced O_2^{-} production, indicating the involvement of plastosemiquinones

in O_2^- production. Because the midpoint redox potentials for tightly bound plastosemiquinones at the Q_A site (E_m (Q_A/Q_A^-) =−60 to −140 mV, pH 7) [29–30] and loosely bound plastosemiquinone at the Q_B site (E_m (Q_B/Q_B^-) =−45 mV, pH 7) [31] are lower than the operational redox potential of O_2/O_2^- redox couple (close to 0 mV or even positive), the reduction of molecular oxygen by plastosemiquinones is feasible. Based on the presented data, we propose that O_2^- is produced by one-electron reduction of molecular oxygen by plastosemiquinones formed by one-electron reduction of plastoquinone at the Q_B sites and one-electron oxidation of plastoquinol at the Q_C site but most likely not the Q_D site (Fig. 5).

1. Involvement of the Q_B site in O_2^- production

In the EPR spin-trapping data obtained using the urea-type herbicide DCMU, the EMPO-OOH adduct EPR signal was only partially suppressed, which indicates that molecular oxygen is reduced prior to the Q_B site (Fig. 2A and B). The DCMU-insensitive EMPO-OOH adduct EPR signal (50%) is likely due to reduction of molecular oxygen by $Pheo^-$ or Q_A^-. It has been previously proposed that $Pheo^-$ and Q_A^- function as the predominant electron donors to molecular oxygen due to their low redox potentials [20, 22, 23, 48]. The DCMU-sensitive EMPO-OOH adduct EPR signal (50%) corresponds to the formation of O_2^- via reduction of molecular oxygen by plastosemiquinone formed at or after the Q_B site. Electron transfer from Q_A^- to loosely bound plastoquinone at the Q_B site yields Q_B^-, which subsequently forms the more stable Q_BH by protonation of proximal amino acids. Subsequent Q_BH reduction and protonation yield Q_BH_2, which moves out through the channels [11]. However, if protonation of Q_B^- by proximal amino acids slows, the lifetime of Q_B^- increases. When molecular oxygen is in the proximity to Q_B^-, reduction of molecular oxygen by Q_B^- produces O_2^-.

2. Involvement of the Q_C site in O_2^- production

Based on X-ray crystal structural analyses of the PSII complex, Q_BH_2 exchange by plastoquinone at the Q_B site was proposed to occur via plastoquinol diffusion through channel I (bottom channel) and II (upper channel) [2]. During this process, Q_BH_2 liberates from the Q_B site and diffuses through the bottom channel to the Q_C site located in the vicinity of the heme iron of cyt b_{559} at distance of 17 Å from the head group of plastoquinol. Plastoquinol binding at the Q_C site was proposed to favour electron donation to the ferric heme iron of cyt b_{559} [46]. Illumination of PSII membranes caused the photoreduction of the HP form of cyt b_{559}, demonstrating that Q_CH_2 is oxidized by the ferric heme iron of cyt b_{559} to form Q_CH. Here, we propose that Q_CH reduces molecular oxygen to O_2^-. Because addition of dinoseb to the PSII membranes partially enhanced O_2^- formation (Fig. 2C), we propose that the ferrous heme iron of LP cyt b_{559} reduces molecular oxygen, which forms O_2^-. Fig. 4C and D) show that the HP form of cyt

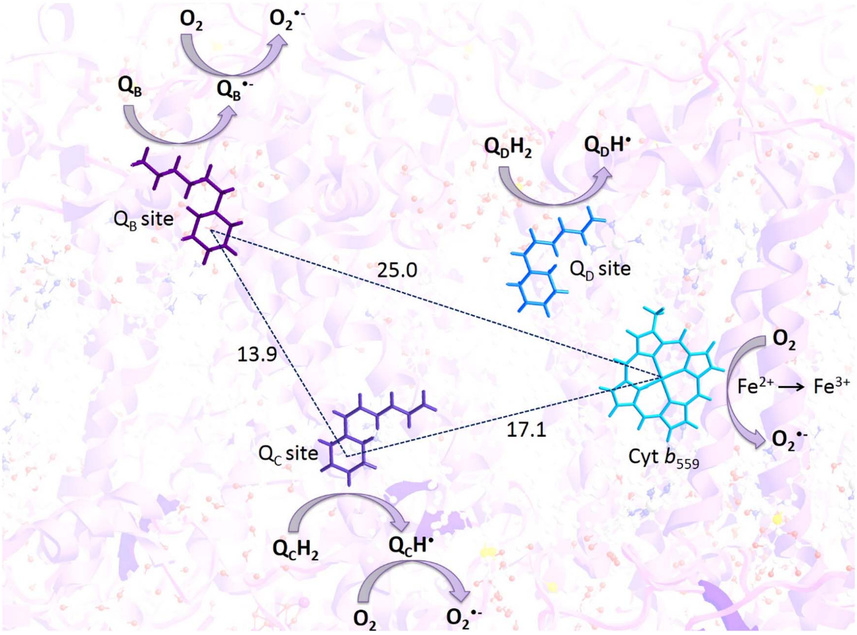

Fig. 5. Proposed mechanism for the involvement of loosely bound plastosemiquinone at the Q_B and Q_C sites in O_2^- formation in PSII. Superoxide anion radicals are produced via one-electron reduction of molecular oxygen by plastosemiquinones, which are formed via one-electron reduction of plastoquinone at the Q_B sites and one-electron oxidation of plastoquinol at the Q_C site but unlikely at the Q_D site.

doi:10.1371/journal.pone.0115466.g005

b_{559} was converted to the LP form in the presence of dinoseb, as previously demonstrated by Kaminskaya and Shuvalov [15]. In addition to binding of dinoseb to Q_D site which has been claimed in the recent past, it is also known to bind to Q_B site. In such a case, the formation of Q_CH is unlikely formed by oxidation of Q_cH_2; however, the alternative reaction pathway for formation of Q_CH occurs. Consistent with this proposal, the formation of Q_CH by one-electron reduction of plastoquinone cannot be excluded [26] and thus the involvement of Q_CH and LP form of cyt b_{559} in O_2^- formation via the Q_C site might be considered.

3. Involvement of the Q_D site in O_2^- production

The observation that the phenolic-type herbicide dinoseb, which binds at the Q_D site enhanced EMPO-OOH adduct EPR signal further indicates that Q_DH formed by plastoquinol oxidation at the Q_D site is not involved in O_2^- production (Fig. 2A). Q_DH_2 oxidation by the heme iron of the HP form of cyt b_{559} and deprotonation by proximal amino acids results in the formation of Q_DH. Kaminskaya and Shuvalov [15] recently suggested that Q_DH is stable at the Q_D site, and the midpoint redox potentials of the Q_D/Q_DH redox couple are more positive than those of the Q_B/Q_B^- redox couple (Em $=-45$ mV, pH 7). Consistent with this proposal, we assume that the reduction of molecular oxygen by Q_DH is not feasible and thus O_2^- formation at the Q_D site is ambiguous.

Conclusion

The data presented in this study demonstrate that loosely bound plastosemiquinones at the Q_B and Q_C sites are involved in the formation of O_2^- via one-electron reduction of molecular oxygen. Loosely bound plastosemiquinone Q_B^- is formed via one-electron reduction of plastoquinone at the Q_B site; however, one-electron oxidation of plastoquinol by cyt b_{559} at the Q_C site forms Q_CH. By contrast, the results indicated that O_2^- formation from plastosemiquinones at the Q_D site was ambiguous. In addition to loosely bound plastosemiquinone, previous studies have reported the formation of O_2^- by free plastosemiquinone in the PQ pool [25, 43–45]. The interaction of plastoquinol with plastoquinone in the PQ pool was suggested to result in the formation of free PQ^-, which reduces molecular oxygen to form O_2^-. Further studies are needed to elucidate a unifying mechanism for O_2^- formation which involves PQ pool.

Acknowledgments

We are grateful to Dr. Jan Hrbáč for his support with respect to the EPR measurements.

Author Contributions

Conceived and designed the experiments: DKY PP. Performed the experiments: DKY AP. Analyzed the data: DKY AP. Contributed reagents/materials/analysis tools: PP. Wrote the paper: DKY PP AP JK.

References

1. **Ferreira KN, Iverson TM, Maghlaoui K, Barber J, Iwata S** (2004) Architecture of the photosynthetic oxygen-evolving center. Science 303: 1831–1838.

2. **Guskov A, Kern J, Gabdulkhakov A, Broser M, Zouni A, et al.** (2009) Cynobacterial photosystem II at 2.9 Å resolution and the role of quinones, lipids, channels and chloride. Nat Struct Mol Biol 16: 334–342.

3. **Umena Y, Kawakami K, Shen J-R, Kamiya N** (2011) Crystal structure of oxygen-evolving photosystem II at a resolution of 1.9 Å, Nature 473: 55–61.

4. **Renger G, Holzwarth AR** (2005) Primary electron transfer in the RC II. In:, Wydrzynski TJ, Satoh K, (eds.,), Photosystem II: the light-driven water: plastoquinoneoxidoreductase, Springer, Dordrecht, , 139–175.

5. **Rappaport F, Diner BA** (2008) Primary photochemistry and energetics leading to the oxidation of the $(Mn)_4Ca$ cluster and to the evolution of molecular oxygen in photosystem II. Coord Chem Rev 252: 259–272.

6. **Brudvig GW** (2008) Water oxidation chemistry of photosystem II. Phil Trans R Soc B 363: 1211–1219.

7. **Cardona T, Sedoud A, Cox N, Rutherford AW** (2012) Charge separation in photosystem II: a comparative and evolutionary overview. Biochim Biophys Acta 1817: 26–43.

8. **Grundmeier A, Dau H** (2012) Structural models of the magnese complex of photosystem II and mechanistic implications. Biochim Biophys Acta 1817: 88–105.

9. **Müh F, Glöckner C, Hellmich J, Zouni A** (2012) Light-induced quinone reduction in photosystem II, Biochim Biophys Acta 1817: 44–65.

10. **Kruk J, Strzałka K** (2001) Redox changes of cytochrome b_{559} in the presence of plastoquinones. J Biol Chem 276: 86–91.

11. **Kaminskaya O, Shuvalov VA, Renger G** (2007) Two reaction pathways for transformation of high potential cytochrome b559 of PSII into the intermediate potential form. Biochim Biophys Acta 1767: 550–558.

12. **Kaminskaya O, Shuvalov VA, Renger G** (2007) Evidence for a novel quinone-binding site in the photosystem II (PSII) complex that regulates the redox potential of cytochrome b_{559}. Biochemistry 46: 1091–1105.

13. **Hasegawa K, Noguchi T** (2014) Molecular interaction of the quinone electron acceptor Q_A, Q_B and Q_C in photosystem II as studied by the fragment molecular orbital method. Photosynth Res 120: 113–123.

14. **Lambreva MD, Russo D, Polticelli F, Viviana S, Antonacci A, et al.** (2014) Structure/Function/Dynamics of photosystem II plastoquinone binding sites. Curr Protein Pep Sci 15: 285–295.

15. **Kaminskaya O, Shuvalov VA** (2013) Biphasic reduction of cytochrome b559 by plastoquinol in photosystem II membrane fragments: Evidence of two types of cytochrome b559/plastoquinol redox equilibria. Biochim Biophys Acta 1827: 471–483.

16. **Pospíšil P** (2009) Production of reactive oxygen species by photosystem II. Biochim Biophys Acta 1787: 1151–1160.

17. **Pospíšil P** (2012) Molecular mechanism of production and scavenging of reactive oxygen species by photosystem II. Biochim Biophys Acta 1817: 218–231.

18. **Vass I** (2012) Molecular mechanism of Photodamage in the photosystem II complex. Biochim Biophys Acta 1817: 209–217.

19. **Frankel LK, Sallans L, Limbach PA, Bricker TM** (2012) Identification of oxidized amino acid residues in the vicinity of the Mn_4CaO_5 cluster of photosystem II: Implications for the identification of oxygen channels within the photosystem II, Biochemistry 51: 6371–6377.

20. **Pospíšil P, Arató A, Krieger-Liszkay A, Rutherford AW** (2004) Hydroxyl radical generation by photosystem II. Biochemistry 43: 6783–6792.

21. **Tiwari A, Pospíšil P** (2009) Superoxide oxidase and reductase activity of cytochrome b_{559} in photosystem II. Biochim Biophys Acta 1787: 985–994.

22. **Ananyev G, Renger G, Wacker U, Klimov V** (1994) The photoproduction of superoxide radicals and the superoxide dismutase activity of photosystem II: The possible involvement of cytochrome b_{559}. Photosynth Res 41: 327–338.

23. **Cleland RE, Grace SC** (1999) Voltametric detection of superoxide production by photosystem II. FEBS Lett 457: 348–352.

24. **Zhang S, Weng J, Tu T, Yao S, Xu C** (2003) Study on the photo-generation of superoxide radicals in photosystem II with EPR spin trapping techniques. Photosynth Res 75: 41–48.

25. **Mubarakshina MM, Ivanov BN** (2010) The production and scavenging of reactive oxygen species in the plastoquinone pool of chloroplast thylakoid membranes. Physiol Plant 140: 103–110.

26. **Pospíšil P, Šnyrychová E, Kruk J, Strzalka K, Nauš J** (2006) Evidence that cytochrome b_{559} is involved in superoxide production in photosystem II: effect of synthetic short-chain plastoquinones in a cytochrome b_{559} tobacco mutant. Biochem J 397: 321–327.

27. **Kato Y, Sugiura M, Oda A, Watanabe T** (2009) Spectroelectrochemical determination of the redox potential of pheophytin a, the primary electron acceptor in photosystem II. Proc Natl Acad Sci 106: 17365–17370.

28. **Klimov VV, Allakhverdiev SI, Demeter S, Krasnovsky AA** (1979) Photoreduction of pheophytin in photosystem II of chloroplasts as a function of redox potential of the medium. Dokl Acad Nauk USSR 249: 227–237.

29. **Shibamoto T, Kato Y, Sugiura M, Watanabe T** (2009) Redox potential of the primary plastoquinone electron acceptor QA in photosystem II from *Thermosynechococcus elongatus* determined by spectroelectrochemistry. Biochemistry 48: 10682–10684.

30. **Krieger A, Rutherford AW, Johnson GN** (1995) On the determination of the redox mid-point potential of the primary quinone acceptor, Q_A, in photosystem II. Biochim Biophys Acta 1229: 193–201.

31. **Hauska G, Hurt E, Gabellini N, Locku W** (1983) Comparative aspects of quinol-cytochrome c/plastocyaninoxidor-eductase. Biochim Biophys Acta 726: 97–133.

32. **Wood PM** (1987) The two redox potential for oxygen reduction to superoxide. Trends Biochem Sci 12: 250–251.

33. **Pospíšil P** (2011) Enzymatic function of cyt b559 in photosystem II. J Photochem Photobiol B 104: 341–347.

34. **Berthold DA, Babcock GT, Yocum CF** (1981) A highly resolved oxygen evolving photosystem II preparation from spinach thylakoid membranes. FEBS Lett 134: 231–234.

35. **Ford RC, Evans MCW** (1983) Isolation of a photosystem II from higher plants with highly enriched oxygen evolution activity. FEBS Lett 160: 159–164.

36. **Wydrzynski T, Inoue Y** (1987) Modified photosystem II acceptor side properties upon replacement of the quinone at the Q_B site with 2, 5-dimethyl-*p*-benzoquinone and phenyl-*p*-benzoquinone. Biochim Biophys Acta 893: 33–42.

37. **Kruk J, Karpinski S** (2006) An HPLC-based method of estimation of the total redox stat of plastoquinone in chloroplasts, the size of the photochemically active plastoquinone-pool and its redox state in thylakoids of *Arabidopsis*. Biochim Biophys Acta 1757: 1669–1675.

38. **Zhang H, Joseph J, Vasquez-Vivar J, Karoui H, Nsanzumuhire C, et al.** (2000) Detection of superoxide anion using an isotopically labeled nitrone spin trap: potential biological applications. FEBS Lett 473: 58–62.

39. **Šnyrychová E, Pospíšil P, Nauš J** (2006) The effect of metal chelators on the production of hydroxyl radicals in thylakoids. Photosynth Res 88: 323–329.

40. **Fufezan C, Rutherford AW, Krieger-Liszkay A** (2002) Singlet oxygen production in herbicide-treated photosystem II. FEBS Lett 532: 407–410.

41. **Arató A, Bondarava N, Krieger-Liszkay A** (2004) Production of reactive oxygen species in chloride- and calcium-depleted photosystem II and their involvement in photoinhibition. Biochim Biophys Acta 1608: 171–180.

42. **Büchel C, Barber J, Ananyev G, Eshaghi S, Watt R, et al.** (1999) Photoassembly of the manganese cluster and oxygen evolution from monomeric and dimeric CP47 reaction center photosystem II complexes. Proc Natl Acad Sci 96: 14288–14293.

43. **Khorobrykh S, Mubarakshina M, Ivanov B** (2004) Photosystem I is not solely responsible for oxygen reduction in isolated thylakoids. Biochim Biophys Acta 1665: 164–167.

44. **Mubarakshina M, Khorobrykh S, Ivanov B** (2006) Oxygen reduction in chloroplast thylakoids results in production of hydrogen peroxide inside the membrane. Biochim Biophys Acta 1757: 1496–1503.

45. **Ivanov B, Mubarakshina M, Khorobrykh S** (2007) Kinetics of the plastoquinone pool oxidation following illumination. Oxygen incorporation into photosynthetic electron transport chain. FEBS Lett 581: 1342–1346.

46. **Sinha RK, Tiwari A, Pospíšil P** (2010) Water-splitting manganese complex controls light-induced redox changes of cytochrome b_{559} in photosystem II. J Bioenerg Biomembr 42: 337–344.

47. **Bondarava N, Gross CM, Mubarakshina M, Golecki JR, Johnson GN, et al.** (2010) Putative function of cytochrome b_{559} as a plastoquionol oxidase. Physiol Plant 138: 463–473.

48. **Frankel LK, Sallans L, Limbach PA, Bricker TM** (2013) Oxidized amino acid residues in the vicinity of Q_A and $Pheo_{D1}$ of the photosystem II reaction center: Putative generation sites of reducing-side reactive oxygen species. PLoS ONE 8 e58042.
